# Adaptable Xerogel-Layered Amperometric Biosensor Platforms on Wire Electrodes for Clinically Relevant Measurements

**DOI:** 10.3390/s19112584

**Published:** 2019-06-06

**Authors:** Lillian B. Hughes, Najwa Labban, Grace E. Conway, Julie A. Pollock, Michael C. Leopold

**Affiliations:** Department of Chemistry, Gottwald Science Center, University of Richmond, Richmond, VA 23173, USA; lillian.hughes@richmond.edu (L.B.H.); najwa.labban@richmond.edu (N.L.); grace.conway@richmond.edu (G.E.C.); jpollock@richmond.edu (J.A.P.)

**Keywords:** biosensors, biomedical, in vitro, in vivo, needle electrode, wire, sepsis, enzyme, electrochemical, platinum black, bodily fluids

## Abstract

Biosensing strategies that employ readily adaptable materials for different analytes, can be miniaturized into needle electrode form, and function in bodily fluids represent a significant step toward the development of clinically relevant in vitro and in vivo sensors. In this work, a general scheme for 1st generation amperometric biosensors involving layer-by-layer electrode modification with enzyme-doped xerogels, electrochemically-deposited polymer, and polyurethane semi-permeable membranes is shown to achieve these goals. With minor modifications to these materials, sensors representing potential point-of-care medical tools are demonstrated to be sensitive and selective for a number of conditions. The potential for bedside measurements or continuous monitoring of analytes may offer faster and more accurate clinical diagnoses for diseases such as diabetes (glucose), preeclampsia (uric acid), galactosemia (galactose), xanthinuria (xanthine), and sepsis (lactate). For the specific diagnostic application, the sensing schemes have been miniaturized to wire electrodes and/or demonstrated as functional in synthetic urine or blood serum. Signal enhancement through the incorporation of platinum nanoparticle film in the scheme offers additional design control within the sensing scheme. The presented sensing strategy has the potential to be applied to any disease that has a related biomolecule and corresponding oxidase enzyme and represents rare, adaptable, sensing capabilities.

## 1. Introduction

Biosensors continue to be of significant interest to the scientific community due to their importance in a variety of fields and applications [1,2,3,4,5]. In the field of medicine, the development of sensors that detect molecules that can indicate the presence or progression of a specific disease remains of particular interest [2,6,7]. Identifying clinically relevant analytes of this nature represents an important technological advancement toward development of in vivo devices capable of continuous, real-time monitoring or in vitro diagnostic tools that can be quickly administered at the bedside on small samples of a patient’s blood or urine [8,9,10,11]. In both cases, effective sensors would provide diagnostic information for physicians at a critical juncture and allow for more effective treatment to be administered more quickly. Biosensors must meet certain performance criteria to be considered effective including an ability to selectively detect a specific compound within a relevant matrix (e.g., specific bodily fluid), sensitivity to provide adequate signal-to-noise (S/N), fast response times, and the propensity for microfabrication into small, mass-produced, and easily used devices [12]. While presenting a powerful diagnostic and monitoring tool for clinicians, the research and development of strategies and materials for constructing these devices introduces a number of challenging issues at the interface of analytical chemistry and materials science [13]. 

A number of individual target molecules with links to medical diagnostics or implications for specific disease conditions are known and several examples are discussed herein. Glucose remains one of the most studied molecules for biosensors as it is critical for diabetes management [8]. Other clinically relevant analytes with existing bodies of literature include uric acid (UA) [14], galactose [15], xanthine [16], sarcosine [17], cholesterol [18] and lactate (Lac) [19]. Briefly, abnormal uric acid (UA) levels have been linked to many diseases such as gout and Lesch–Nyhan syndrome; while, more specifically, hyperuricemia is a known indicator of pregnancy-induced hypertension (PIH), a condition that can lead to a dangerous disorder called preeclampsia that poses significant health risks for both mother and child [14,20,21]. The presence of galactose in newborns serves as a primary marker for galactosemia, a potentially lethal autosomal recessive disorder [22,23]. High concentrations of xanthine in urine correspond to a hereditary condition called xanthinuria that can lead to renal failure and kidney stones [16]. Increased levels of sarcosine have been reported as an indicator for prostate cancer that is more reliable than traditional prostate-specific antigen (PSA) testing [24,25]. Sepsis, the body’s extreme and toxic response to infection, represents a continuing problem across the medical field as the condition is often undiagnosed or misdiagnosed by physicians, resulting in nearly 6 million deaths world-wide each year [26,27,28]. A 2004 case study from Nguyen and coworkers [29] identified lactate clearance as a predictive parameter for sepsis progression, which could allow for more accurate diagnosis and early intervention. In all these examples, the diagnostic molecules indicative of the disease or condition also have an associated enzymatic oxidation reaction for their metabolism.

Electrochemical biosensors, more specifically, 1st generation amperometric biosensors, represent a type of sensing platform that offers a number of the desired attributes for these biomedical sensors [30]. First, these biosensing schemes achieve selectivity because they harness enzymatic reactions at electrodes, detecting the analyte indirectly via the oxidation of hydrogen peroxide (H_2_O_2_), a natural by-product of the enzyme-catalyzed reaction between the target substrate and oxygen—a relatively simple detection mechanism [30,31]:

(1) $$\mathrm{Substrate}+O2\overset{\mathrm{Enzyme}}{\to }H_{2}O_{2}+\mathrm{products}$$

First generation amperometric biosensors are pursued for medical applications because they also offer affordability, simplicity, and, in theory, are amenable to miniaturization [6,7] As such, researchers have explored a plethora of customized 1st generation amperometric biosensing schemes targeting clinically relevant molecules and examples in the literature are plentiful. While the reports are numerous, it is import to note that each scheme is typically elaborately designed for a particular analyte and most often constructed at a macroelectrode platform with the reported potential to eventually miniaturize the scheme to a microelectrode, though actual transition to the smaller platform is often only theoretically envisioned in many of the studies [1,2,4,32,33]. The demonstration of the functionality of a sensing scheme after being scaled down is a critical research milestone toward realizing effective in vivo implants or in vitro devices. In the former case, smaller sensor size allows for minimizing inflammatory response to a foreign material, thereby improving biocompatibility [34]. Sensors miniaturized to a scale of less than 0.5 mm, smaller than the typical size of syringe needles and catheters, are of particular interest [34,35,36]. Miniaturized biosensing schemes must retain the sensitivity and selectivity effectiveness demonstrated on macroelectrode platforms—a non-trivial objective. 

**Scheme 1:**
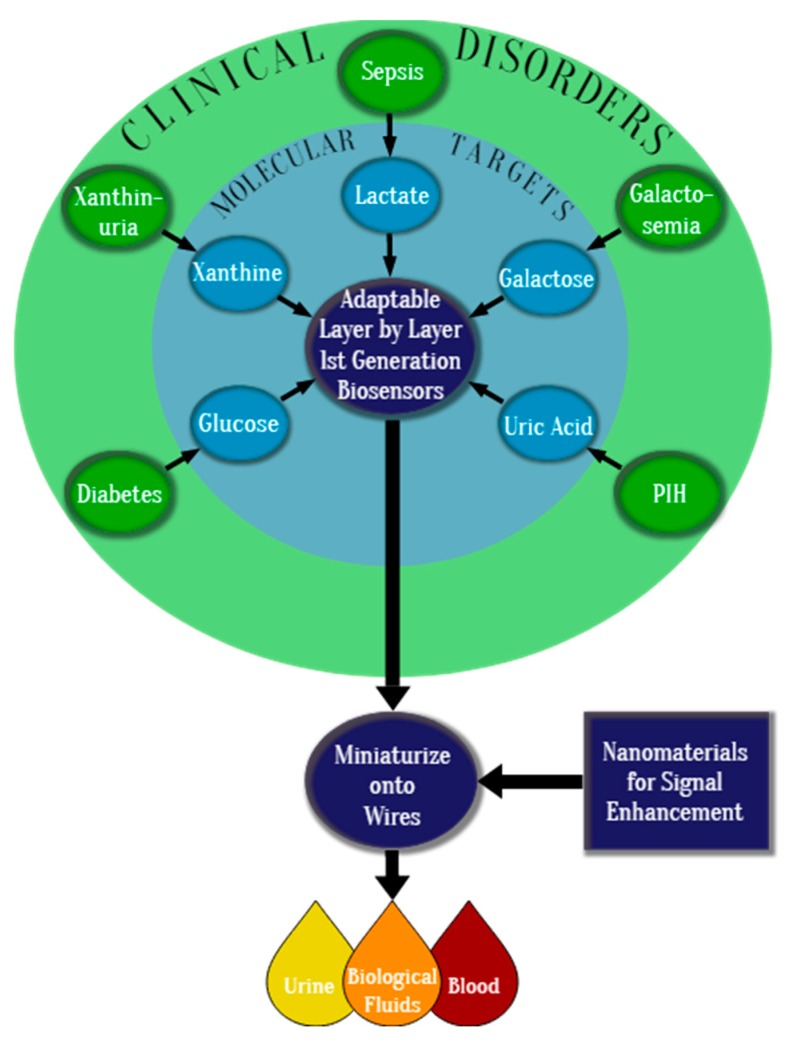
Schematic representation of strategy for biosensor development for medical applications.

Other critical elements employed in the construction of such sensors is the methodology and materials used to immobilize the enzyme at the electrode surface. One strategy for sensor design that has emerged involves modifying the electrode with multiple layers of functional materials in what is known as layer-by-layer (LbL) methodology [37,38]. While numerous materials such as electropolymers [39], hydrogels [40] and nanoporous gold [41], for example, have been exploited for this purpose, a seminal report by Bright and coworkers in 1994 examined an LbL scheme incorporating solution–gel (sol–gel) materials as a functional component of a glucose biosensor [42]. Sol–gels are networks of polymerized silanes that provide a three-dimensional scaffolding for multi-layers of immobilized enzyme at an electrode, forming a xerogel with solvent evaporation [43]. Enzyme microencapsulation within xerogel matrices yields several well-established advantages, including mild synthetic conditions preserving the structure and function of the embedded enzymes, chemical inertness, physical rigidity, negligible swelling in aqueous solutions, thermal/photochemical stability, and synthetically tunable porosity, a property of particular interest for biosensing structures [44]. Prior work in our own group has explored LbL construction of xerogel films as part of 1st generation amperometric biosensors for glucose [3,45], uric acid [12] and galactose [22]—all on macroelectrode platforms. While these systems were all robust in performance and, in some cases, demonstrated the use of nanoparticle networks to enhance signal [45,46], we noted that they employed similar LbL methods and materials. The similarity in the approaches suggested that it might be possible to present a general strategy and set of materials as a type of template. This general template would allow for targeting a number of different compounds with only minor adaptions of the layers and a scheme amenable to both miniaturization to microelectrodes and operation in relevant bodily fluids (Scheme 1). 

In this work, we explore a general template for the LbL construction of xerogel-based 1st generation amperometric biosensors on needle microelectrodes that are able to target multiple disease-related analytes within the bodily fluids most relevant for the particular disease marker. While some prior work in this area has demonstrated the miniaturization of LbL systems containing enzyme-doped xerogel schemes onto needle-type electrodes [34,36,47,48,49,50], those designs are specific for a singular analyte and do not investigate a versatile strategy to be used for multiple target species with only moderate adaptations. Herein, we demonstrate the successful adaptations of both known and previously unreported LbL xerogel systems to needle-type electrodes and show their functionality in relevant bodily fluids. The incorporation of nanoparticle films within the same general sensing scheme at the needle electrodes is also explored as a means to enhance amperometric signal. While we were able to demonstrate these abilities with a number of different systems shown in Scheme 1, we continue to use glucose and glucose oxidase as a model system while also highlighting two previously unreported systems, lactate and xanthine detection, to illustrate the adaptability of the template. While many reports highlight the use of sol-gel materials [44], LbL approaches [37,38] and/or nanomaterials [1,13,32] in the sensing of a particular analyte, the systematic expansion of a general scheme to multiple targets is rarer. The demonstrated versatility and functionality of the presented biosensors both adapted to wire electrodes and operational in relevant biological matrices, represents important steps toward the realization of bioanalytical sensors capable of continuous in vitro clinical measurements, as well as future devices with in vivo capability. 

## 2. Experimental Details

### 2.1. Materials and Instrumentation 

Unless otherwise stated, all chemicals were reagent grade or higher and purchased from Millipore-Sigma (Burlington, MA, USA). Tecoflex SG-80A polyurethane (TPU) and Hydrothane AL25-80A polyurethane (HPU) were obtained from Lubrizol (Wickliffe, OH, USA) and AdvanSource Biomaterials (Wilmington, MA, USA), respectively. A platinizing solution (3% chloroplatinic acid, v/v in water) was purchased from Lab-Chem (Pittsburgh, PA, USA). Synthetic urine (Surine) was obtained from Cerilliant (Sigma-Aldrich) and blood serum (sheep) from Hemostat (Dixon, CA, USA). All other solutions were made with 18 MΩ·cm ultra-purified water (UP H_2_O) (PureLab-Ultra, Elga, High Wycombe, UK). Teflon (PTFE)-coated platinum:iridium (70:30) wire (Sigmund-Cohn, Medwire, Mt. Vernon, NY, USA) with a diameter of 245 µm (330 µm coated) or pure platinum wire (A-M Systems, Sequim, WA, USA) with a diameter of 127 µm (203 µm coated) were used as wire working electrodes (WE) while 2 mm diameter (CH Instruments, Austin, TX, USA) platinum electrodes served as macroelectrode WEs. In either case, the analytical performance of the sensors were evaluated via amperometric current–time (I–t) curves recorded on 8-channel potentiostats (CH Instruments, 1000B or 1030C) or a single channel potentiostat (CH Instruments, 420B). Electrochemistry was measured versus a common Ag/AgCl (saturated KCl) aqueous reference electrode (RE) (CH Instruments) and platinum wire counter electrode. Silanes for xerogel fabrication were purchased from Sigma-Aldrich (octyl-trimethoxysilane (OTMS), isobutyl-trimethoxysilane (IBTMS), amino-propyl-trimethoxysilane (APTMS), 3-mercaptopropyl-trimethoxy silane (MPTMS)) or from Gelest, Inc. (Morrisville, PA, USA), hydroxymethyl-triethoxysilane (HMTES)) and were stored in a desiccated glovebox (Plas Labs, Inc., Lansing, MI, USA) and transported using sealed/desiccated microcentrifuge tubes to exclude moisture until deposition of the material as a film. Silane bottles were replaced every 3–4 weeks in case of inadvertent hydrolysis. 

### 2.2. Enzymes

Glucose oxidase (*Aspergillus niger*), galactose oxidase (*Dactylium dendroides*), and xanthine oxidase (bovine milk) were purchased from Millipore-Sigma. Urate oxidase or uricase from *Bacillus fastidiosus* was purchased from Millipore-Sigma or produced in-house [12] as previously described, Additional uricase (*Candida utilis*, recombinant), lactate oxidase (*Pediococcus* sp.), and xanthine oxidase (native microorganisms) was purchased from Creative Enzymes^®^ (Shirley, NY, USA). 

### 2.3. Fabrication of Layered Sol-gel Based Wire Biosensors

A ~5 cm length of PTFE-insulated Pt-Ir wire was cut and ~4 mm of the end exposed by removal of the PTFE coating to create the WEs. The bare sensor was electrochemically cleaned via cyclic voltammetry (−0.25 to +1.20 V, 0.25 V s^−1^) in 0.1 M H_2_SO_4_ until the voltammetry was representative of a clean platinum surface. After cleaning, wire WEs were modified via specific but similar procedures depending on their intended targets. The following example for the glucose model system is provided, with additional details of similar procedures for the other sensors provided in the Supplementary Materials. 

In brief, for the glucose biosensors on wire electrodes, glucose oxidase (GOx) doped sol-gels of OTMS were formed by first dissolving 9.0 mg of GOx in 75 μL of UP H_2_O in a centrifugation vial and, in a separate vial, diluting 25 μL of OTMS with 100 μL of tetrahydrofuran (THF). These vials were sealed and hand-vortexed vigorously for 5 min and 1 min, respectively. After individual mixing, 50 μL of the GOx/H_2_O solution was transferred to the OTMS/THF mixture and shaken for an additional 1 min to facilitate the formation of a sol−gel. The process of deposition of the sol-gel onto the wire electrode was adapted from Schoenfisch et al [47], coating with the GOx precursor solution by dip-coating the sensor five times (5 s submerged time, 10 s dry period under ambient conditions). The electrodes were then dried horizontally for 30 min at 50% RH (relative humidity) before the next sol–gel deposition. The second layer of sol–gel was prepared as above with the omission of GOx, following the same dip-coating procedure to provide a diffusion-limiting layer. Unless otherwise stated, sol−gel coated electrodes were allowed to form xerogels over 48 h inside a temperature/50% relative humidity (RH) controlled chamber (Cole-Parmer, Vernon Hills, IL, USA) [45,51]. Notice: Strict RH control, through the use of desiccated glove boxes/vessels during storage/handling of silanes as well as with a humidity-controlled chamber during deposition/drying of the sol−gels was found to be critical for consistent sensor performance [45].

After application of the sol-gel layers, an additional polymer layer was added. For example, for glucose sensing, a polyphenol layer was applied to assist the selectivity of the sensors [47,52,53]. A solution of phenol was prepared (25 mL, 40 mM) with 60 mM PBS at pH = 7.4, degassed with N_2_ for 20 min and electropolymerized onto the wire sensor by chronocoulometry (held at +0.9 V for 15 min while stirring). Sensors were rinsed with UP H_2_O and allowed to dry horizontally for 30 min at 50% RH. An outer polyurethane (PU) layer was then deposited in order to limit diffusion and improve glucose selectivity [45,47]. A 50:50 PU blend of hydrophilic HPU and hydrophobic TPU was prepared by adding 50 mg of HPU and TPU each to a 5 mL solution of THF/EtOH (50:50 v/v) and stirred overnight. The PU was dip-coated on the wire electrodes for 10 cycles as described above and were allowed to dry for 30 min horizontally at 50% RH. The tip of the sensor was capped with epoxy and allowed to dry for 45 min at 50% RH, resulting in a 3–4 mm sensing pseudo-cavity. A similar detailed procedure for the other biosensors can be found in the Supplementary Materials, including uric acid, lactate, and galactose, for examples. Additionally, fabrication of the sensors on macroelectrodes proceeded in a similar fashion with the exception that prior to electrochemical cycling to clean the electrode, the macroelectrodes were polished with successively smaller alumina on a polishing wheel and rinsed with copious amounts of water [3]. 

### 2.4. Platinum Black Modification of Wire Electrodes

Prior to sol–gel deposition, cleaned bare electrodes were platinized in 3% chloroplatinic acid (v/v in water) by cycling the potential from +0.6 to −0.35 V at a sweep rate of 20 mV s^−1^ to obtain a Pt-B-modified platinum working electrode [46,54]. The Pt-B-modified surface was assessed using scanning electron microscopy (SEM) imaging and surface area was calculated with well-established chronocoulometry methodology [55,56].

### 2.5. Evaluation of Biosensor Performance

Completed sensors were soaked in 60 mM potassium phosphate buffer solution (PBS) at pH 7.4 for a minimum of 1 h to reduce current drift and impregnate the sol−gel with buffer solution. Most biosensors were subjected to +0.65 V in 25 mL of PBS for at least 20 min prior to injection of any substrate/analyte to stabilize sensor reading. Sensors were calibrated by adding successive injections of increasing concentrations of substrate. 

## 3. Results and Discussion

**Figure 1:**
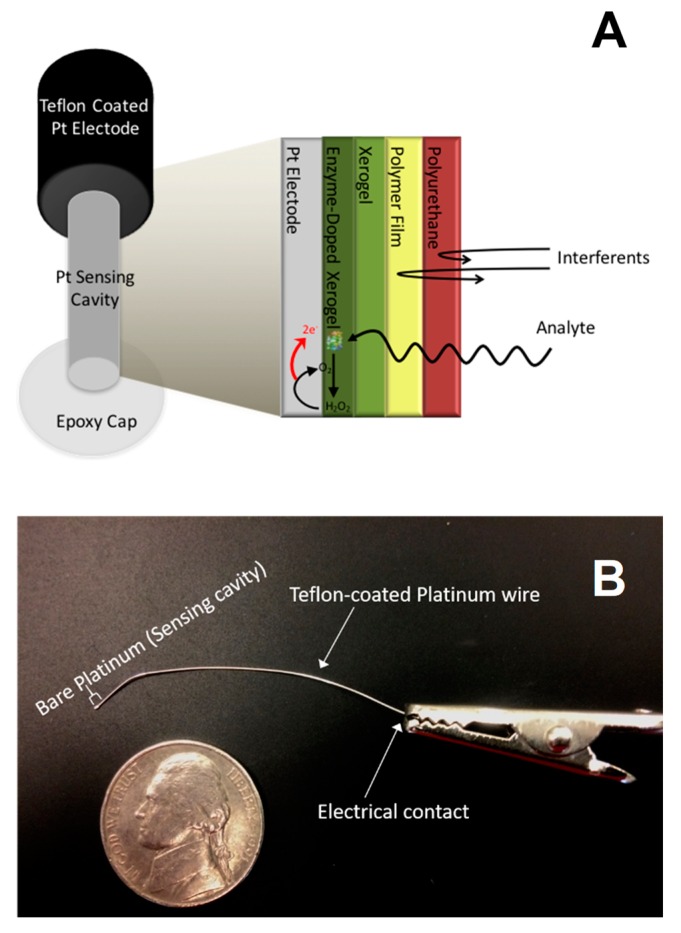
(**A**) Generic LbL scheme at the wire electrode interface that can feature some combination of materials such as enzyme-doped and/or undoped xerogel, electrochemically deposited semi-permeable polymers, and polyurethane layers; (**B**) photograph of 127 μm diameter platinum wire with exposed tip for LbL coatings.

The basic design of the biosensing scheme includes some combination of four layers of electrode modification: (1) an enzyme-encapsulating xerogel layer, (2) an undoped xerogel (diffusional layer), (3) an inner selective electrochemically-deposited polymer layer, and (4) a multi-functional ad-layer of polyurethane (Figure 1A) [3,12]. Each of the layers had been previously characterized and studied for their properties, including entire reports on electrochemically-deposited polymer films and work establishing that the xerogel porosity was controllable based on moisture content, aging time, and silane precursor. Moreover, specific combinations of this type of layering were previously investigated and optimized for the detection of target analytes at the macroelectrode surfaces, including successful glucose [3], uric acid [12] and galactose [22] biosensors. In those studies, it was shown that each layer serves a specific and functional purpose, whether enhancing sensitivity, improving selectivity against interferents, or preventing enzyme leakage. The challenge of the current study was to successfully transition this scheme to needle electrodes while showing it to be versatile enough to be applied to a number of different targets, including some systems not previously explored. Additionally, as part of the scheme’s versatility, signal enhancement strategies using nanoparticle films were explored as an option for the scheme and operational viability in relevant bodily fluids was also investigated. With these goals, however, it is important to note that one must consider the ultimate sensing goals for a particular system. For example, while miniaturization might be useful for in vivo device development (e.g., continuous, real-time implant for lactate measurements), it may not be necessary for some in vitro applications (e.g., sarcosine prostate cancer screening or galactose testing for galatosemia testing). Similarly, operation in specific bodily fluids may be more important for specific analytes compared to others. If the target is expected to be present in urine, it is unnecessary to show operational viability for detection of the species in other matrices (e.g., blood serum). It is with these considerations that the following studies were conducted. However, as in prior studies [45] and given its affordability and availability, the glucose/glucose oxidase (GOx) system continued to serve as a practical target/enzyme model system for initial, proof-of-concept experiments. 

### 3.1. Miniaturization of Model Scheme onto Needle-Type Electrode

Miniaturization of a biosensor presents a challenge in that, as one significantly changes the size and shape of the transducer, there is no guarantee that the sensing response achieved on macroelectrodes will be effective on a smaller platform with different geometry. The LbL strategy using xerogel materials was employed to construct sensors at the miniaturized electrochemical interface of the exposed tip of a Teflon-coated platinum wire (Figure 1B). In transitioning these sensing systems to a wire geometry with a diameter in the range of 120–250 μm, a small area at the end of the coated platinum wire (<3–4 mm) was first exposed via removal of the Teflon coating, electrochemically cleaned, and subsequently modified as shown in Figure 1A. In this study, for proof-of-concept operation of these sensors at the wires, an epoxy cap at the tip of the wire was used to form a pseudo sensing cavity where the film was constructed and minimize sensing irregularities that might arise from the otherwise exposed and irregular geometry of the cross-section of the wire’s tip (Figure 1B). Once films were formed in the pseudo cavities, the wire-based sensors were evaluated on sensing performance similar to macroelectrode systems, including measurements of sensitivity, response time, and selectivity. 

**Figure 2:**
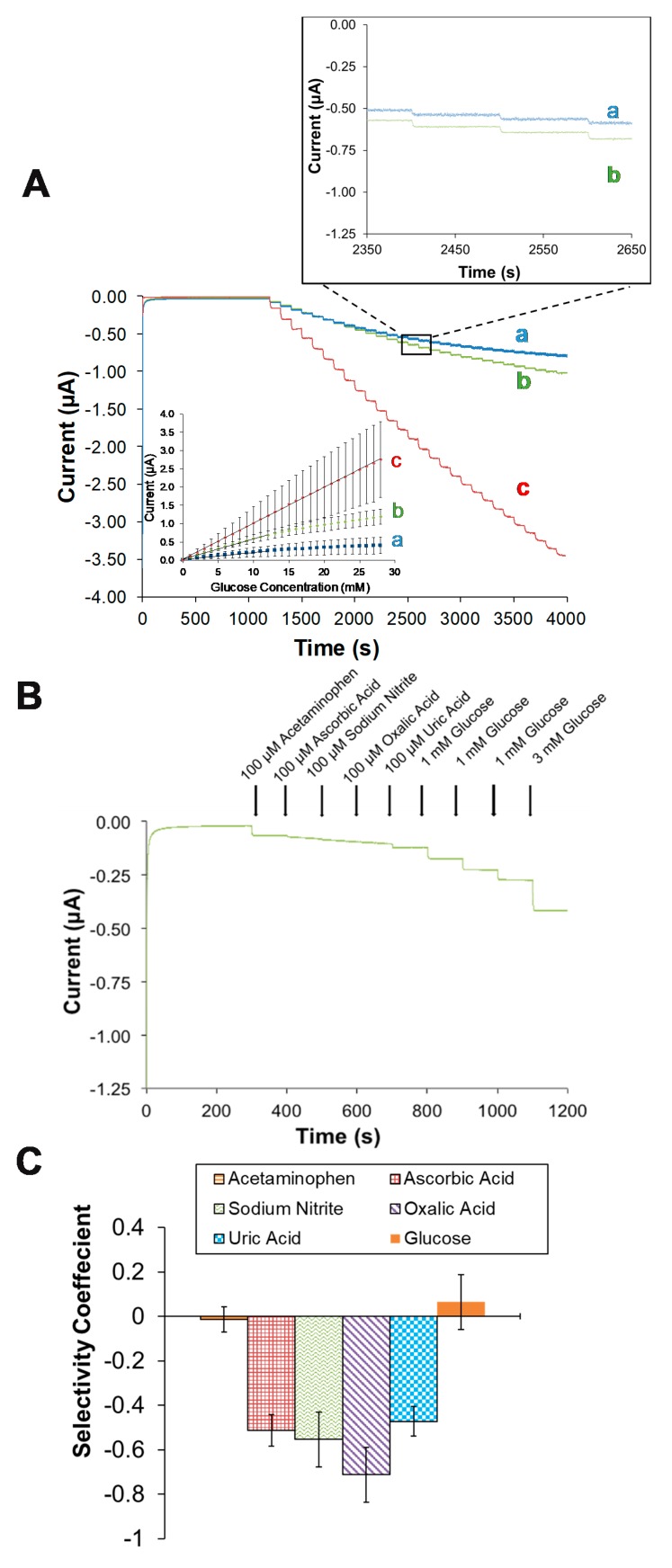
(**A**) Typical amperometric I-t response and corresponding calibration curve (inset) during successive 1 mM injections of glucose at a (a) Pt wire electrode, (b) Pt-Ir wire electrode, and (c) Pt macroelectrode modified with GOx-doped OTMS xerogel, undoped OTMS xerogel, polyphenol (PP), polyurethane layer (50:50), and epoxy cap; (**B**) Typical amperometric I-t response and (**C**) calculated selectivity coefficients during successive injections of common interferent species (100 μM) and glucose (1 mM and/or 3 mM) at a Pt-Ir wire electrode modified with the same four layers described in (A). Notes: Soln. is 60.0 mM PBS, pH = 7.4, μ = 155 mM (wires) and 4.4 mM PBS, pH = 7.0 (macroelectrode). Note: Error bars represent standard error (n ≥ 3).

Miniaturization of a xerogel-based glucose biosensor was first explored as a model system, adapting fabrication methods from prior procedures used with macroelectrodes that produced a highly effective sensing apparatus [3,45]. The specific scheme, consisting of a GOx-doped and undoped xerogel, inner selective polymer, and outer 50:50 blend polyurethane coating constructed at both the platinum-iridium wires (d = 245 µm) as well as the relatively smaller (Figure 1B) pure platinum wire (d = 127 µm). The analytical sensing performance at the modified wire electrodes, including the dynamic/linear range of the amperometric step response and sensitivity, was compared with analogous glucose systems at macroelectrodes (Figure 2A). Each system exhibited a well-defined, stair-step response to glucose injections that translates to a dynamic range from 0 to 28 mM and linear calibration curves for all three electrode geometries that easily span the physiologically relevant concentrations of glucose (~1–10 mM) [2]. While smaller linear ranges are noted for the wire systems, the resemblance of their stepping response to the macro system suggests the robustness of the sensor design despite its minimized size. Sensor-to-sensor variability is observable in the results, an expected consequence of multiple people, hand-constructing four-layers of materials at the various electrode geometries. 

A significant challenge of most biosensor designs is achieving sufficient sensitivity while simultaneously discriminating against common interferent species. Both the electrochemically-deposited polymer and PU layers have been previously shown to aid in selectivity and were similarly incorporated within the miniaturized glucose sensing scheme and tested for interferent response [3,57]. Figure 2B shows an I-t curve of the same OTMS xerogel systems on platinum-iridium wire (d = 245 µm) electrodes capped with both polyphenol and 50:50 PU designed for glucose sensing during injections of common interferents (i.e., acetaminophen, ascorbic acid (AA), sodium nitrite, oxalic acid, and uric acid) as well as standard glucose injections of different concentrations. As seen in Figure 2B, current responses from the injection of endogenous physiological interferents are nearly negligible compared to those of the glucose injections. Only acetaminophen registers a significant measurable response and, while this represents a challenge, it is important to remember that acetaminophen is a synthetic pharmaceutical that is ingested and clears the body in a relatively short time (4–6 h). Thus, in any eventual medical application of these sensors, the presence of acetaminophen should be identifiable with an effective patient history [55]. Selectivity coefficients (K_amp_) were calculated to conservatively and quantitatively assess selectivity as has been done in previous studies [47,55] using the following equation:

(2) $$K_{j}^{\mathrm{amp}}=\log\;\left(\frac{∆I_{j}/C_{j}}{∆I_{g}/C_{g}}\right)$$

where ∆I_j_ and ∆I_g_ are the measured currents for a specific interferent species (j) and glucose and C_j_ and C_g_ are concentrations of the interferent species and glucose, respectively. Negative values of K_amp_ indicate that the interferent is inconsequential (i.e., no significant response compared to analyte response) whereas species with near zero or positive values are selected for by the sensor. In this case, the magnitude of the K_amp_ values (Figure 2C) suggest excellent discriminatory selectivity of the sensing scheme even at a miniaturized interface. A similar I-t curve establishing selectivity against most interferent species was achieved at the smaller platinum wires as well (Supplementary Materials, Figure SM-1). 

It is important to note that the mechanical removal of the wire coating and subsequent application of the epoxy cap to the tip of the wire results in a variable electroactive surface area for each sensor. As such, the model glucose system was used to confirm that this variation in area does not affect the overall response linearity of these miniaturized sensors. The electrochemical determination of individual wire areas allows for a comparison of a current response versus a current density response at the wire electrodes. Specifically, chronocoulometry of potassium ferricyanide at the wire electrode was used to assess the electroactive surface area of the pseudo cavity prior to modification and normalize current response to electrode area [24]. An example of the chronocoulometric analysis for electroactive surface area is shown in the Supplementary Materials (Figure SM-2). Linearity in the calibration curves between 0 and 12 mM glucose was maintained regardless of area standardization (Supplementary Materials, Figure SM-3) demonstrates the dependability of the scheme design despite wire-to-wire geometrical variations. Based on this result, with regard to the wire microelectrodes, the remainder of our study herein was conducted exclusively with the platinum-iridium wires with a bare diameter of 245 μm. 

### 3.2. Versatility of Scheme for Multiple Analytes at Needle-Type Electrodes

As previously mentioned, most biosensing schemes found in literature reports are elaborately constructed to target a single analyte and cannot be easily adapted to a different target. A major proposed advantage of the strategy and materials utilized with the presented scheme is that it can be readily adapted to both a wire geometry and a variety of different analytes. For this study, adaptations of the four-layer scheme (Figure 1A) previously reported only at macroelectrodes were transitioned to wire electrodes while new, previously unreported systems were also targeted for adaptation to wires. 

#### 3.2.1. Uric Acid and Galactose Biosensors

**Figure 3:**
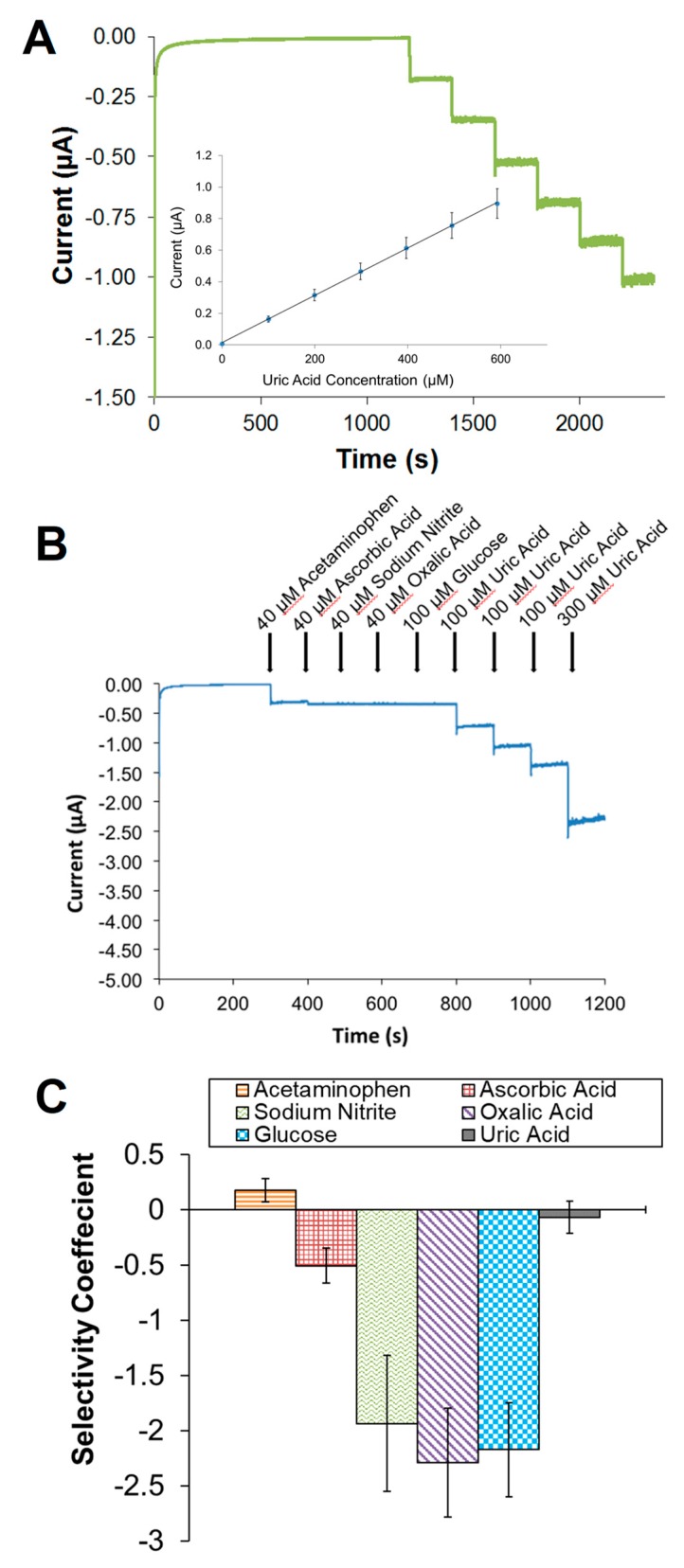
(**A**) Typical amperometric I-t response and calibration curve (inset) during successive 100 μM injections of uric acid at a Pt-Ir wire electrode modified with UOx-doped HMTES xerogel, undoped HMTES xerogel, polyluminol-polyaniline (PL-A), and polyurethane layer (100% HPU), and epoxy cap; (**B**) Typical amperometric I-t response and (**C**) calculated selectivity coefficients during successive injections of common interferent species (40 μM) and uric acid (100 μM and/or 300 μM) at a Pt-Ir wire electrode modified as in (A); Solution: 60.0 mM PBS, pH = 7.4, μ = 155 mM. Note: Error bars represent standard error (n ≥ 10).

Elevated levels of uric acid (UA) or hyperuricemia can serve as a reliable diagnostic marker for pregnancy-induced hypertension (PIH), a disorder that can lead to a more serious, life-threatening, condition called pre-eclampsia [12]. A uric acid biosensor, particularly one that can be miniaturized for potential real-time measurement devices would allow for faster and more accurate diagnosis of patients at risk for these complications. Wire electrodes were modified with uricase (UOx) doped HMTES xerogel, followed by an undoped layer of HMTES and subsequently capped with a polyluminol-polyaniline (PL-A) electrochemically-deposited polymer layer and polyurethane (100% HPU), the same layering strategy successfully applied to macroelectrodes in a previous study [3,47]. As shown in Figure 3A, the modified as described wires exhibited a robust stair-step response to increasing concentrations of uric acid and produced a calibration curve with linearity through the relevant physiological range (100 to 600 μM). As in with the glucose model system, the uric acid wire sensors reported excellent discrimination against common, naturally occurring interferent species (Figure 3B,C). Only acetaminophen results in a significant current response and again, as an artificial interferent whose consumption can be reported by patients, presents less of a concern than other species. 

A similar successful transition to wire electrodes was achieved for LbL-constructed galactose biosensor that was recently reported at macroelectrodes [22]. Accurate galactose measurements in newborns can predict the presence of the disorder galactosemia [23]. Based on the prior report at macroelectrodes [22] wire electrodes were modified with GaOx-doped IBTMS xerogel, a 50:50 PU layer and an epoxy cap (Figure 1A). As with the UA adaptation, a stair-step amperometric I-t response was achieved and translated to a linear calibration curve with a physiologically relevant linear range up to 7 mM galactose and fast response times (Supplementary Materials, Figure SM-4). In both cases, UA and galactose biosensing, comparisons of the sensor performance with literature reports of sensors for the same targets can be found in the original studies [12,22]. 

#### 3.2.2. Xanthine Biosensors

While the previously presented results focus on systems that were previously established at macroelectrodes, it was also of interest to see if the strategy and materials could be adapted to previously unreported and more complex systems. The detection of xanthine is critical for diagnosing xanthinuria in humans and can also serve as an indicator for potential hand-held sensors able to detect spoiled meat and fish [16]. In terms of biosensing, xanthine presents a unique situation for a LbL system given the enzymatic reaction of xanthine oxidase (XOx) with the xanthine produces H_2_O_2_ and uric acid (UA)—the ratio of which is dependent on the amount of oxygen present: [58].

(3) $$\mathrm{Xanthine}+O2\overset{\mathrm{Xox}}{\to }H_{2}O_{2}+\mathrm{uric}\;\mathrm{acid}$$

The products are important in this case because both are electroactive and will be oxidized at an electrode held at the typical +0.65 V. In order to apply our materials and LbL strategy, it is critical to first completely understand the components of the amperometric signal observed. As such, when both H_2_O_2_ and UA are directly injected at an electrode poised at +0.65V they each yield a significant amperometric response (Supplementary Materials, Figure SM-5A). However, if the potential is held at +0.35 V, H_2_O_2_ oxidation is observed while the UA oxidation is selectively eliminated (Supplementary Materials, Figure SM-5B). A similar experiment was conducted with XOx-doped PTMS xerogel and capped with PU (100% HPU) and subjected to injections of xanthine. At an electrode held at +0.65 V, the injections of xanthine result in a stair-step response, which is attributed to both H_2_O_2_ and UA oxidation. The presence of both species is confirmed with the injection of a significant amount (1 μM) of catalase, an enzyme that consumes H_2_O_2_. The catalase injection causes an abrupt decrease in current but does not return the amperometric response to baseline, suggesting the continuing presence of UA product in the solution. However, if the signal from UA is turned off by applying a potential of +0.35 V as to isolate only the H_2_O_2_ oxidation, a similar, but significantly smaller stair-step response is observed. In this case, injection of catalase returns the signal nearly to baseline (Supplementary Materials, Figure SM-6). In this manner, we can stabilize xanthine sensing response by understanding and controlling the species creating the analytical signal. 

Prior to testing on wire electrodes, and as with other systems tested, the four-layer scheme (Figure 1A) was first applied to a platinum macroelectrode platform. Platinum electrodes modified with XOx-doped PTMS xerogel and PU capping layer (100% HPU) were tested for xanthine sensitivity and selectivity. A typical I-t curve during xanthine injections and the corresponding calibration curve, which easily spans the relevant physiological range (0–100 μM) shows that the system is sensitive for the detection of xanthine. Similarly, the amperometric response during interferent and xanthine injections, along with corresponding calculated selectivity coefficients, support that the adaptation to xanthine is also produces selective response (Supplementary Materials, Figure SM-7). The performance of the xanthine sensor is comparable to reports in the literature for other xanthine sensing systems [59,60,61,62,63,64,65] compiled and provided in Supplementary Materials (Table SM-1). 

**Figure 4:**
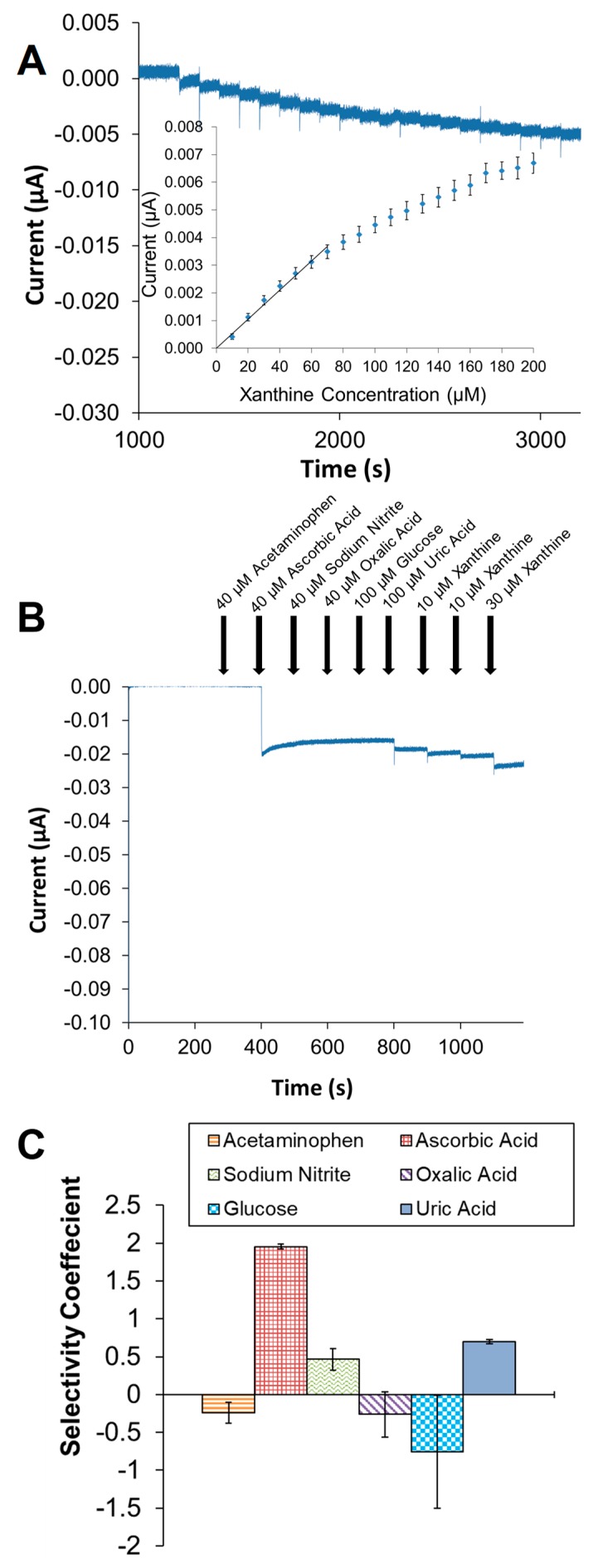
(**A**) Typical amperometric I-t response and calibration curve (inset) during successive 10 μM injections of xanthine at a Pt-Ir wire electrode (+0.35V) modified with XOx-doped PTMS xerogels, polyurethane layer (100% HPU), and epoxy cap. (**B**) Typical amperometric I-t response and (**C**) calculated selectivity coefficients during successive injections of common interferent species (40 μM and/or 100 μM) and xanthine (10 μM and/or 30 μM) at a Pt-Ir wire electrode (+0.35V) modified as in (A); Solution: 60.0 mM PBS, pH = 7.4, μ = 155 mM. Note: The error bars represent standard error (n ≥ 10).

With the verified ability to differentiate UA response from H_2_O_2_ signal via the applied voltage, the system was readily adapted to wire electrodes. The same materials comprising the composite film at the macroelectrode were applied to the wire electrode with the results shown in Figure 4. The films at wire electrodes, though exhibiting an expectedly lower current response, showed definitive stair-step responsiveness toward xanthine injections, a substantial linear range, and similar response time (Figure 4A). Selectivity toward xanthine was largely maintained at the wire electrode (Figure 4B,C) though the different electrode geometry appears to allow for a greater AA interferent response compared to the macroelectrode system—a result suggesting that future research efforts may focus on identifying an appropriate electrochemically-deposited polymer film to exclude that particular species [53]. While the rigorous measurement of selectivity coefficients for the modified wire electrode (Figure 4C) suggests that sodium nitrite (NaNO_2_) is registering a substantial interferent response, particularly compared to the macroelectrode system (Supplementary Materials, Figure SM-7B), we note from the actual and typical I-t response at the wire (shown in (Figure 4B) that there is no significant signal generated upon injection of NaNO_2_. These results suggest that the selectivity coefficient for NaNO_2_ is likely artificially inflated due to the rising background current observed after the substantial AA response. 

#### 3.2.3. Lactate Biosensors

One of the most consequential developments of our strategy and scheme would be an adaptation to lactate detection as it relates to tools for sepsis diagnosis and monitoring. Sepsis is a systemic response of a body to serious infection. In the U.S. alone, more than 1.5 million people get sepsis every year [27], resulting in approximately 250,000 deaths (5000 children’s deaths) [66], while worldwide, an estimated 30 million people contract the condition [67] including 6 million newborns [28]. One in three patients in the United States who develops sepsis will die in the hospital from the condition—making it the leading cause of deaths in hospitals in this country [27]. Accurate diagnosis of sepsis and early intervention at the emergency room with broad spectrum antibiotics, intravenous fluids, and, if possible, bacterial specific antibiotics (after blood testing) represent life saving measures. Unfortunately, sepsis is often undetected or commonly misdiagnosed and, if untreated, very quickly transitions from general sepsis to severe sepsis and eventually to septic shock where corresponding tissue hypoxia and organ failure render a >50% mortality rate [27,28,29]. A number of recent cases in the news have illustrated the danger of misdiagnosis with sepsis. The 2012 case of Rory Staunton, a 12-year-old boy who developed sepsis after getting a cut at his gym and died only 3 days later, has increased awareness of sepsis through The Rory Staunton Foundation for Sepsis Prevention but also continuously updates with victims of sepsis [68]. Additional sepsis cases have recently received high media attention, including the death of a 29-year-old mother of seven after a minor skin infection [69] and a 31-year-old man succumbing to sepsis after swimming with a new tattoo [70]. In very recent news, Jakelin Maquin, age 7, died of septic shock while in the custody of the U.S. Border Patrol [71]. 

Current sepsis diagnosis procedures still primarily rely on physical examination (e.g., fever, heart rate, breathing), which is prone to misdiagnosis as a flu-like illness, or time-consuming laboratory tests (e.g. X-ray, blood and urine analyses) during which common sepsis can progress toward septic shock with significantly increased mortality. A 2004 case study [29] identified lactate clearance (LC) as a predictive parameter for sepsis survival where out of more than 100 patients presenting with severe sepsis/septic shock symptoms, those with elevated LC (>10%) after 6 hours were determined to have a significantly higher probability of survival. Patients exhibiting persistent low levels of LC were associated with an almost 90% mortality rate. The study suggests that lactate, particularly if it could be continuously monitored with an in vivo implantable device or a bedside in vitro quick test, may be an effective tool for early and accurate diagnosis of sepsis, allowing physicians to administer more effective treatment of sepsis. 

As with the other systems, LbL-constructed modification of a macroelectrode in order to establish lactate sensitivity and selectivity preceded attempts to miniaturize to wire electrodes. Pt macroelectrodes were modified with an electropolymerized pyrrole layer as a selective membrane prior to deposition of a HMTES xerogel co-embedded with both lactate oxidase (LOx) and bovine serum albumin (BSA), the latter serving as a stabilizing agent as in prior reports utilizing LOx [72]. The HMTES xerogel was capped with PU (100% HPU). The system responded to 1 mM injections of sodium lactate, yielding the expected stair-step amperometric response typical of these systems (Supplementary Materials, Figure SM-8A). Unlike other systems, however, the lactate sensor shows a strong initial amperometric response during early injections of lactate before becoming more consistent and eventually dissipating. The reasons for this inconsistency are not fully understood and it does result in a two stage calibration curve that is linear in both the low or normal (i.e., resting) physiological lactate concentration range (<3 mM) as well as the elevated or abnormal (>3 mM) range common to sepsis conditions (Supplementary Materials, Figure SM-8A, inset). Interferent testing of this system was also successful with the layered sensor showing excellent selectivity for lactate and discrimination against all interferents except acetaminophen, the only non-naturally occurring interferent (Supplementary Materials, Figure SM-8B). The performance of the lactate sensor is comparable to other reports in the literature describing similar types of electrochemical sensors [39,72,73,74,75,76,77,78,79,80] (Supplementary Materials, Table SM-2).

**Figure 5:**
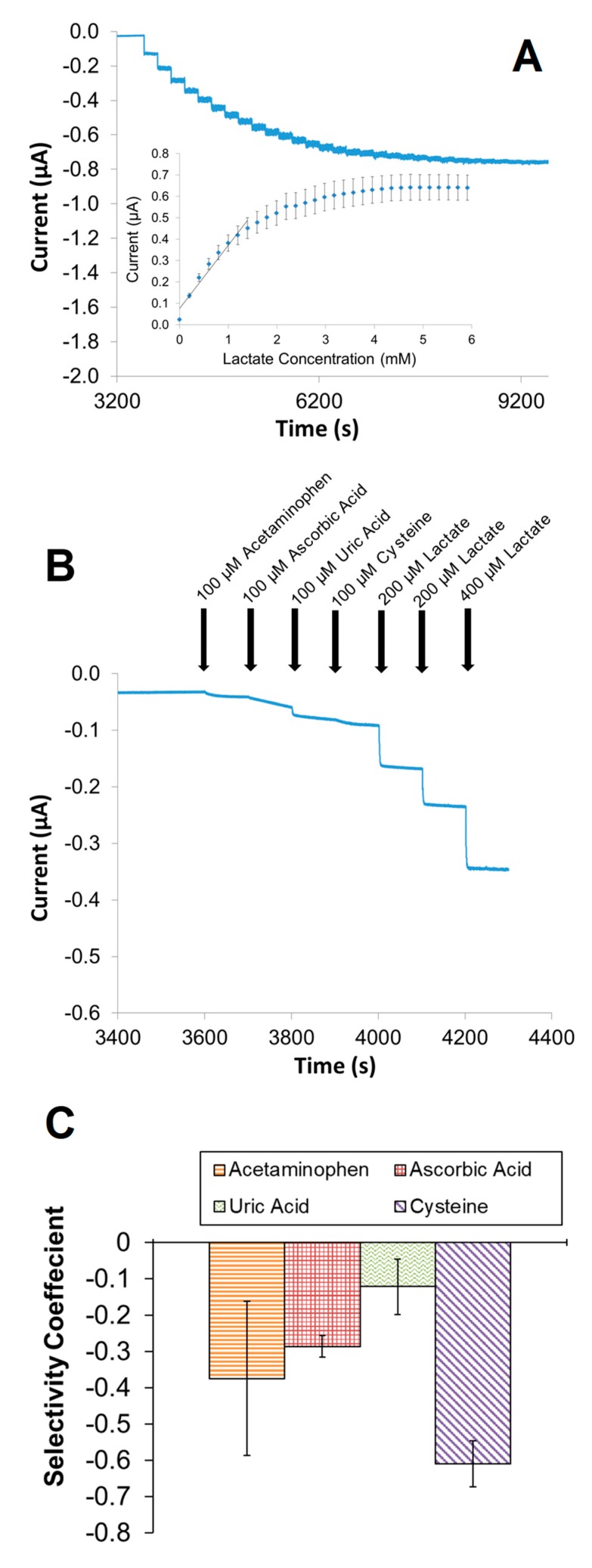
(**A**) Typical amperometric I-t response and calibration curve (inset) during successive 200 μM injections of lactate at a Pt-Ir wire electrode (+0.65 V) modified with polypyrrole, LOx and BSA co-doped HMTES xerogels, polyurethane layer (100% HPU), and epoxy cap. (**B**) Typical amperometric I-t response and (**C**) calculated selectivity coefficients during successive injections of common interferent species (100 μM) and lactate (200 μM and 400 μM) at a Pt-Ir wire electrode modified as in (A); Solution: 60.0 mM PBS, pH = 7.4, μ = 155 mM. Note: The error bars represent standard error (n ≥ 3).

As with the other systems after successful demonstration of functionality of the LbL construction and materials at macroelectrode platforms, it was then transitioned to wire electrodes. The adaptation of the lactate detection scheme to a wire electrode was particularly critical given that the targeted application could involve the insertion of a needle sensor capable of real-time lactate measurement into the flesh of an emergency room patient as is already done with an intravenous line in current practice. Figure 5 illustrates the successful transition of the system to the wire electrode. As with the other systems, a fast, stair-step response with a substantial linear range is achieved prior to the onset of Michaelis-Menton kinetic limitations (Figure 5A) [22,51]. Additionally, the film system maintains effective selectivity toward lactate with nearly negligible responses toward major interferents, including acetaminophen, AA, UA, and cysteine (Figure 5B), a result reinforced in the calculation of negative selectivity coefficients for those particular interferents (Figure 5C). The successful demonstration of the versatility of the materials and the general strategy suggests that both could readily be applied to additional species not currently under investigation where an enzyme-catalyzed reaction metabolizes a target molecule related to a specific molecule that is a diagnostic marker for a medical condition (e.g., cholesterol for heart disease [18] sarcosine for early prostate cancer detection [17,24]).

### 3.3. Signal Enhancement Strategies within the LbL Design

Given the established versatility of the scheme toward different analytes, it is important to note that the proper detection of any particular target species by a sensor will be closely related to their physiologically relevant concentration range. In some cases, detection of any significant concentration of analyte is diagnostically significant (e.g., galactose [23], sarcosine [24]) while in other situations the concentration ranges needing to be monitored can vary over different range (e.g., normal glucose 4–7 mM [2], normal uric acid 200–500 μM [12]). The ability to adjust sensor sensitivity toward an analyte via the introduction of a simple tool that is also amenable to incorporation into the wire scheme would be a distinct advantage of the LbL strategy that has been demonstrated. Additionally, in cases where an in vivo implant for real-time monitoring of an analyte is desired, the sensor design in this study would likely also need to be coupled to biocompatibility strategies that often include additional layering such as NO releasing materials [81]. All additional layers of material represent a trade off in adding functionality at the cost of depressing signal. As such, additional layers supplementing the scheme (Figure 1A) may allow the signal enhancement needed to reach a physiological range of analyte concentration or provide signal enhancement if used in conjunction with biocompatibility strategies.

**Figure 6:**
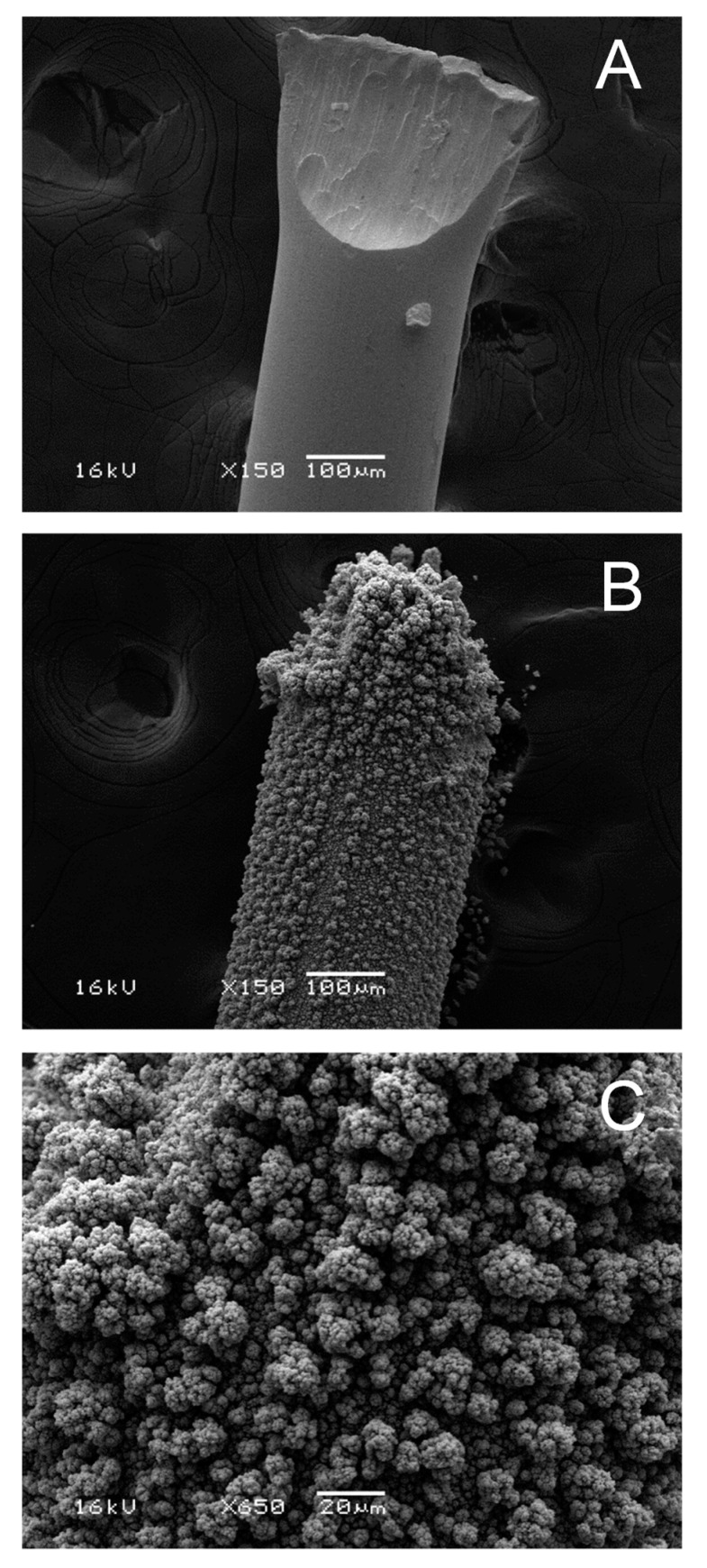
SEM images of Pt-Ir wire (exposed, cut tip) both (**A**) before and (**B**) after modification of Pt black including (**C**) magnified image showing morphology of Pt black. Note: Size bars in each image represent 100 µm, 100 µm, and 20 µm respectively.

For the wire-type electrodes in this study, it remains desirable to improve the signal without sacrificing the miniaturization—that is, improving the electroactive or real surface area without significantly increasing the geometric area. To achieve this, the application of an under-layer of platinum black (Pt-B), shown previously to improve sensitivity at macroelectrodes [46] was electrochemically applied via cyclic voltammetry to the wire systems (see Experimental). The application of Pt-B material results in a visible change in the appearance of the wire, transitioning from a shiny metallic luster of platinum to a black coating (Supplementary Materials, Figure SM-9). Figure 6 shows SEM imaging of the wire and illustrates the morphology changes accompanying the modification of the wire electrode with Pt-B, a process that both visually and experimentally, as measured with chronocoulometry (Supplementary Materials, Figure SM-2), clearly increases the electroactive surface area without significant change to the overall geometric area. 

**Figure 7:**
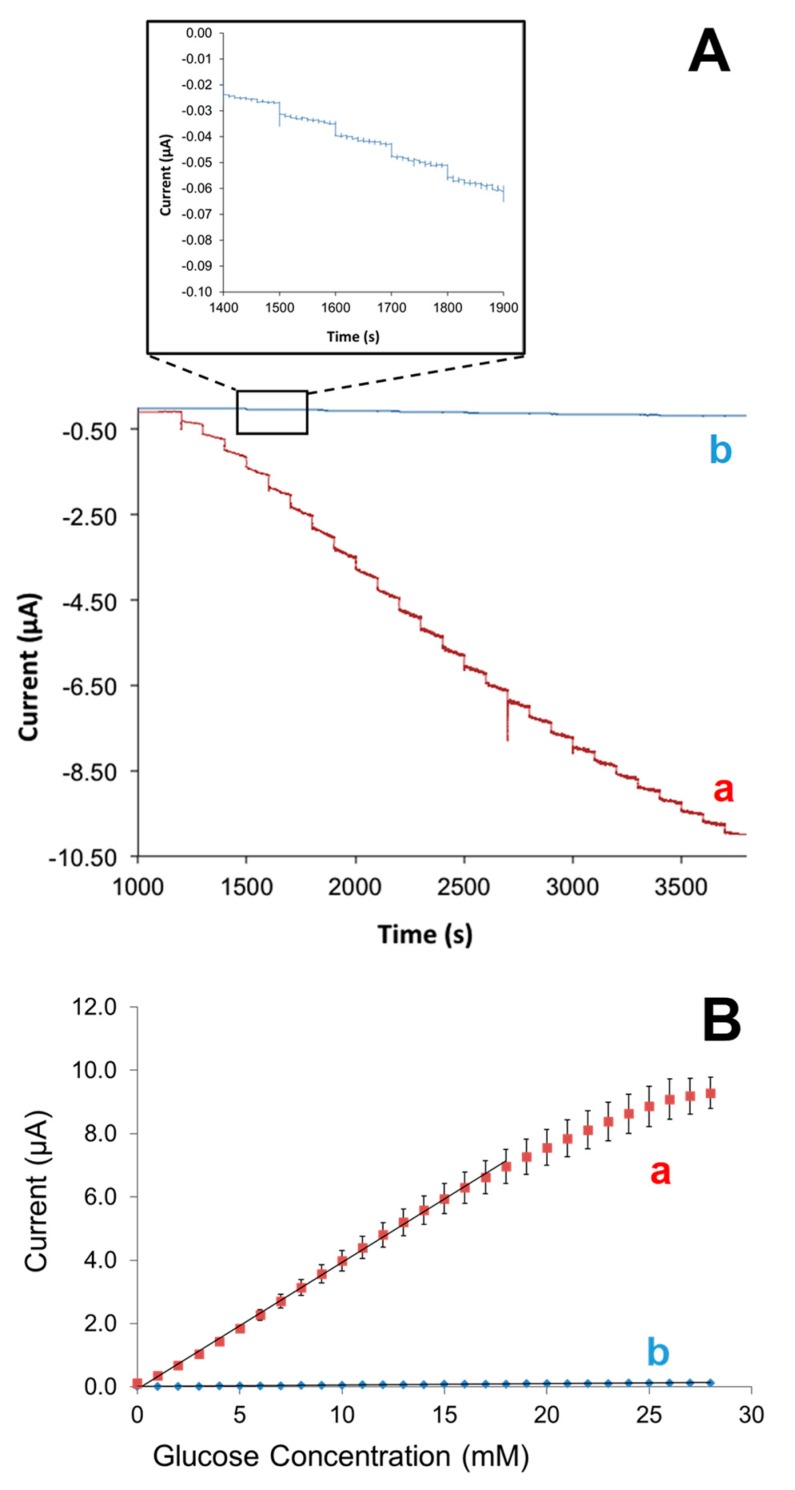
(**A**) Amperometric I-t curves and (**B**) corresponding calibration curves during successive 1 μM injections of glucose at a Pt-Ir wire electrode modified with GOx-doped OTMS xerogel, undoped OTMS xerogel, polyphenol, polyurethane layer (50% HPU, 50% TPU), and epoxy cap both (**a**) with and (**b**) without a Pt-black under-layer. Solution: 60.0 mM PBS, pH = 7.4, μ = 155 mM. Note: The error bars represent standard error (n ≥ 4).

The Pt-B layer can be applied under the initial xerogel layer with ease and has a substantial signal enhancement effect. Figure 7 illustrates the typical enhancement observed with the addition of the Pt-B under-layer—shown for the glucose model system at a wire. The results clearly indicate a much more substantial, larger current stair-step response is achieved with the use of the Pt-black under-layer—a response that translates into a calibration curve with a substantially higher slope (i.e., higher sensitivity toward glucose). The effect of adding Pt-B under-layer is substantial in every scheme in which it was tested. This robust form of signal enhancement through the use of Pt nanoparticle films was demonstrated on a number of issues, including a uric acid, LbL constructed wire electrode (Supplementary Materials, Figure SM-10). The enhancement is anticipated to be effective for all of the schemes presented.

### 3.4. Operation of Wire Electrode Sensors in Relevant Bodily Fluids

The demonstration of these schemes and materials being operational in relevant bodily fluids is a critical step in achieving greater versatility. While some sensors can be applied to buffered solutions, it is important that, when necessary or applicable for a specific diagnostic, they function in systems like blood, blood serum, or urine. As with the other forms of versatility, macroelectrodes modified with the LbL scheme for the detection of glucose, our model system, were tested in both synthetic urine and blood serum solutions. The stair-step amperometric response to increasing concentrations of glucose were again evident for both types of media (Supplementary Materials, Figure SM-11). Additionally, similar responses were achieved for LbL modification of a macro platinum electrodes designed for either UA or xanthine detection in blood serum and synthetic urine (Supplementary Materials, Figures SM-12, SM-13). In both cases, the background or charging current (i.e., non-Faradaic signal) was observed to be larger in the blood serum samples. Given the substantial presence of electrolyte and interferent species as well as the higher viscosity of serum compared to buffered solution or synthetic urine samples, this artifact of the results is unsurprising. 

**Figure 8:**
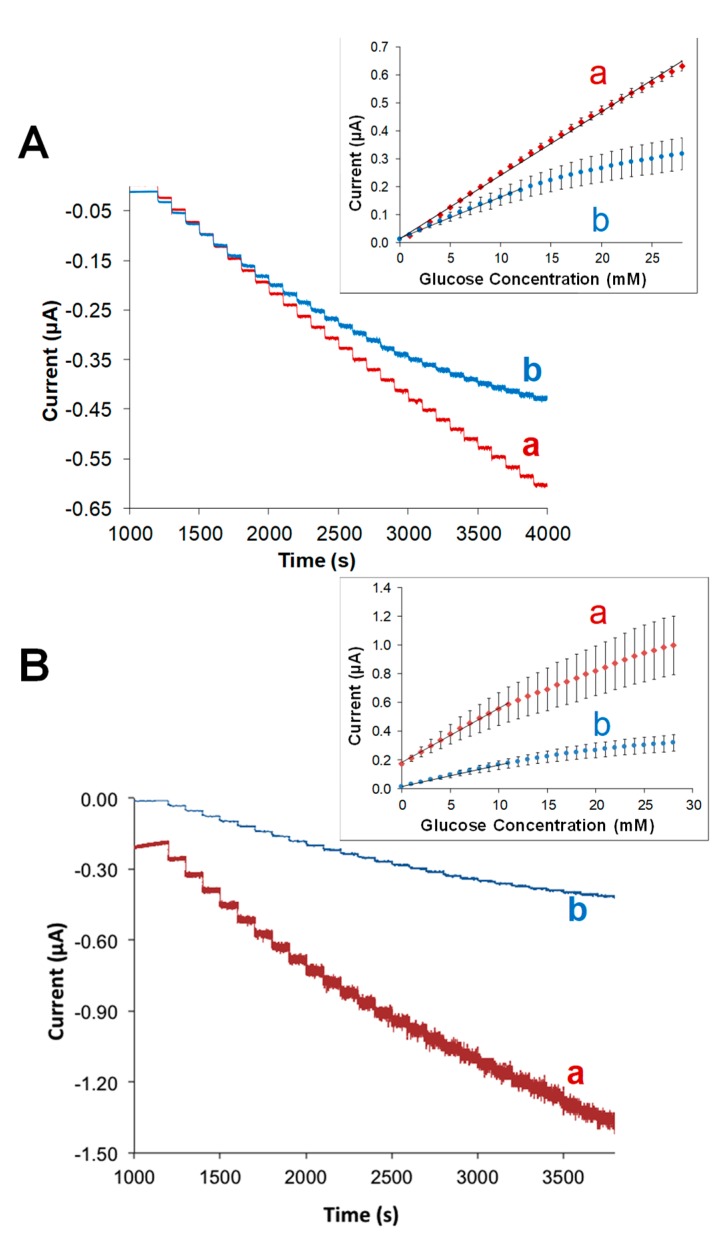
Amperometric I-t curves and corresponding calibration curves (insets) during successive 1 mM injections of glucose at (**A**) Pt macroelectrodes (**a**) vs. Pt-Ir wire electrodes (**b**) each modified with GOx-doped OTMS xerogel, undoped OTMS xerogel, polyphenol (PP), and polyurethane layer (PU) tested in synthetic urine; (**B**) The same LbL system at Pt-Ir wire electrodes (a) with and (b) without Pt-black underlayer and tested in synthetic urine. Note: Error bars represent standard error (n ≥ 3).

Wires were successfully modified with the same LbL films for the glucose model system and were demonstrated to be operational in bodily fluids. For example, Figure 8A shows the amperometric I-t stair-step response and corresponding calibration curves for the modified macroelectrode versus the modified wire electrode in synthetic urine media. While some of the linear range is attenuated with the wire electrode, the calibration curve is still linear through the physiologically relevant concentration range. Additionally, the signal can be successfully enhanced in the same media with the use of a Pt-B under-layer as part of the modified Pt-Ir wire electrode (Figure 8B). While the Pt-B increases charging current, consistent with increased electroactive area, it clearly enhances the current in the synthetic urine media and yields a calibration curve with a greater slope. These results suggest viability of the system on wires and within this bodily fluid with a viable option to enhance signal with a Pt-black under-layer. A similar result was achieved with the same system in blood serum as well (Supplementary Materials, Figure SM-14). 

**Figure 9:**
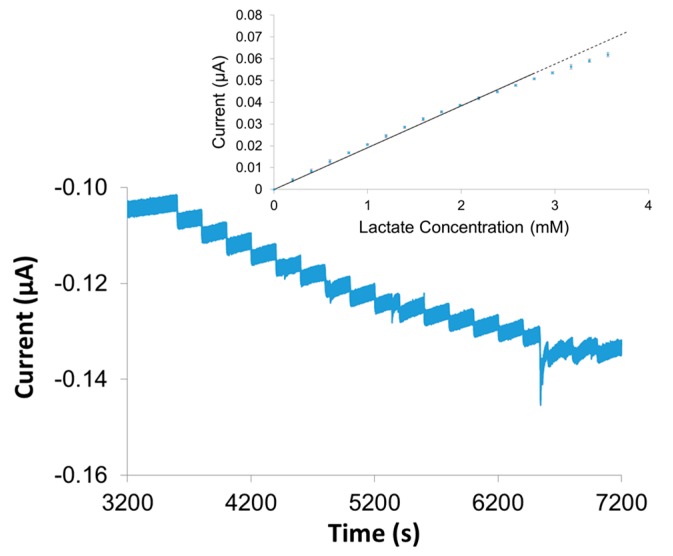
Typical amperometric I-t curve and corresponding calibration curve during successive 0.2 mM injections of lactate at Pt-Ir wire modified with electrochemically-deposited pyrrole layer, LOx and BSA-doped HMTES xerogel, polyurethane layer (100% PU) capping layer, and epoxy cap tested in blood serum. Notes: Amperometric responses have been normalized to compensate for rising background charging current; error bars represent standard deviation of the mean (n ≥ 3).

One of the most important sensing schemes to demonstrate as functional within relevant bodily fluids is the lactate sensor as it is one of the more likely systems to eventually be developed into a real-time, continuous measurement in vivo sensor for sepsis diagnosis. For this specific application of the sensors clinicians would be less concerned with determining a blood concentration and more interested in monitoring lactate clearance over time (i.e., changing concentration) [29]. LbL construction of the LOx system was applied to wire electrodes and tested in blood serum with the results shown in Figure 9. The stair-step response upon injection of lactate to the serum is clearly observed and translates to a significant linear range in the calibration curve (Figure 9, inset). While gaining better control over the charging current background remains an area of interest during further development of these sensors, the results clearly establish the viability and promise of the overall strategy with the successful sensing of one of the more challenging target molecules within a bodily fluid matrix. 

## 4. Conclusions

This work has demonstrated the high functionality and impressive adaptability of LbL film assemblies featuring xerogel layers with immobilized enzymes with electrochemically-deposited polymer layers and polyurethane-based selective membranes utilized for clinically relevant 1st generation amperometic biosensors. While the primary versatility of the general sensing scheme stems from the ability target a wide range of disease-related targets via minor modifications of the layering material, assembly procedure, or testing parameters, it also includes adaptation of the scheme to wire/needle electrodes, a critical step for both small sample testing at the bedside (i.e., in vitro measurements) or continuous intravenous measurements with in vivo implants. Specifically, the wire electrodes here have been adapted easily and effectively for the detection of five analytes including glucose, galactose, uric acid, xanthine, and lactate implicated in their respective diseases of diabetes, galactosemia, PIH, xanthinuria, and sepsis. The approach presented is bolstered by the option to utilize nanomaterials to enhance signal and the demonstration that the systems remain viable in bodily fluids. While the study focused on modifying the tip of a needle electrode which would be easily damaged or disrupted if used in an in vivo application, the strategy and materials of the sensing scheme should be easily amenable to a sensing cavity on the stem of beveled needles to better protect the sensor (Supplementary Materials, Figure SM-15). Similarly, the LbL modification schemes of the working electrodes in this report, particularly the wire electrodes, are compatible with established methodology incorporating an integrated reference electrode on the same wire as well as biocompatibility strategies/materials [8,47]. The demonstrated versatility, miniaturization, and functionality of this general sensor template sets the foundation for achieving sensors that, in theory, can be readily adapted to a large number of clinically relevant targets and is an important advancement toward that goal. 

## Supplementary Materials

The following are available online at https://www.mdpi.com/1424-8220/19/11/2584/s1, Experimental details for fabrication and performance testing of lactate, xanthine, and uric acid sensors; Figure SM-1: I-t curves for GOx system at different-sized wires; Figure SM-2: chronocoulometry for determining electroactive area; Figure SM-3: I-t and calibration curves with and without area standardization (wires); Figure SM-4: I-t and calibration curves for GaOx system at Pt-Ir wire; Figure SM-5: I-t curves for UA and H_2_O_2_ injections; Figure SM-6: I-t curve for xanthine injections with and without catalase; Figure SM-7: I-t and calibration curves with interferent testing for XOx system; Table SM-1: Comparison of sensing performance parameters with literature (XOx system); Figure SM-8: I-t and calibration curves for LOx system; Table SM-2: Comparison of sensing performance parameters with literature (LOx system); Figure SM-9: Cyclic voltammetry for Pt-black formation; Figure SM-10: I-t and calibration curves for Pt-black effect (UOx system); Figure SM-11: I-t and calibration curves for GOx system in blood serum and urine (wire); Figure SM-12: I-t and calibration curves for UOx system in blood serum and urine (wire); Figure SM-13: I-t and calibration curves for XOx system in blood serum and urine; Figure SM-14: I-t and calibration curves for GOx system in blood serum and urine (wire).
