# Supplementary material for: Adaptable Xerogel-Layered Amperometric Biosensor Platforms on Wire Electrodes for Clinically Relevant Measurements
Source: Sensors (Basel). 2019 Jun 6;19(11):2584. doi: 10.3390/s19112584 (PMC6603663; doi:10.3390/s19112584)
Supplement: Supplementary file 1 [file sensors-19-02584-s001.pdf]

Supplementary Materials -

Adaptable Xerogel-Layered Amperometric Biosensor Platforms on Wire Electrodes for Clinically Relevant Measurements

Lillian B. Hughes,† Najwa Labban,† Grace E. Conway, Julie A. Pollock, and Michael C. Leopold*

Department of Chemistry, Gottwald Center for the Sciences, University of Richmond, Richmond, Virginia, 23173

Contents

- Detailed Experimental Details for sensor fabrication and performance testing (wires and macroelectrodes) for lactate, xanthine, and uric acid sesnors - pages 2-4.
- **Figure SM-1.** Amperometric I-t curve during inteferent testing for GOx system on Pt wire (d = 127 μm).
- **Figure SM-2.** Chronocoulometry methodology for determining electroactive area of electrodes with $K_3Fe(CN)_6$.
- **Figure SM-3.** Amperometric I-t and corresponding calibration curves (GOx system) with and without electrode area standardization.
- **Figure SM-4.** Amperometric I-t and corresponding calibration curve for GaOx system on Pt-Ir wire.
- **Figure SM-5.** Amperometric I-t during H_2O_2 and UA injections at bare Pt electrode (+0.65 vs. +0.35 V).
- **Figure SM-6.** Amperometric I-t during xanthine injections at XOx system with catalase injections in solution (+0.65 vs. +0.35 V).
- **Figure SM-7.** Amperometric I-t and corresponding calibration curve with interferent testing for XOx system at platinum macroelectrode.
- **Table SM-1.** Xanthine Biosensor Performance Parameters – Literature Comparison
- **Figure SM-8.** Amperometric I-t and corresponding calibration curve with interferent testing for LOx system at platinum macroelectrode.
- **Table SM-2.** Lactate Biosensor Performance Parameters – Literature Comparison
- **Figure SM-9.** Example of cyclic voltammetry during platinization to form Pt-black and photographs of wire electrodes with and without Pt-black modification.
- **Figure SM-10.** Amperometric I-t and corresponding calibration curve for UOx system at Pt-Ir wire with and without Pt-black underlayer.
- **Figure SM-11.** Amperometric I-t and corresponding calibration curve for GOx system at Pt-Ir wire in blood serum and synthetic urine.
- **Figure SM-12.** Amperometric I-t and corresponding calibration curve for UOx system at Pt macroelectrode in blood serum and synthetic urine.
- **Figure SM-13.** Amperometric I-t and corresponding calibration curve for XOx system at Pt macroelectrode in blood serum and synthetic urine.
- **Figure SM-14.** Amperometric I-t and corresponding calibration curve for GOx system at Pt-Ir wire with and without Pt-black underlayer in blood serum and synthetic urine.
- **Figure SM-15.** Schematic illustration of sensor application via a cavity of a beveled needle.

* To whom correspondence should be addressed. Email: mleopold@richmond.edu: Phone: (804) 287–6329 (MCL).
† These authors contributed equally to this work.

Lactate Sensor Fabrication and Performance

Macroelectrodes. For the lactate biosensors on macroelectrodes, a polypyrrole layer was first applied to assist the selectivity of the sensors. A solution of pyrrole was prepared (25mL, 0.1M) with 0.1mM H_2SO_4, degassed with N_2 for 20 minutes and electropolymerized onto the platinum sensors while stirring using cyclic voltammetry (0.0 to +0.9V, 5 cycles, 50mVs^{-1}). Sensors were allowed to dry for 45 minutes under ambient conditions before being set with sol-gels.

Lactate oxidase (LOx) and bovine serum albumin (BSA) co-doped sol-gels of HMTES were formed by first dissolving 0.8mg of LOx and 0.8mg of BSA in 75µL of UP H_2O in a centrifugation vial and, in a separate vial, mixing 10µL of HMTES with 40µL of NaOH (0.1M). These vials were sealed and machine-vortexed for 10 minutes. After individual mixing, 20µL of the enzyme solution was transferred to the HMTES/NaOH mixture and vortexed for an additional 10 minutes to facilitate the formation of a sol-gel. Electrodes were coated by dropcasting 3µL of the resulting sol-gel onto the pyrrole-modified Pt surface. Sol-gel coated electrodes were allowed to form xerogels over 24h at 50% RH.

An outer layer of polyurethane (PU) was then deposited in order to limit diffusion and improve selectivity. PU was prepared by adding 100mg of HPU to a 5mL solution of THF/EtOH (50:50 v/v) and stirred overnight. The PU was dropcast (10µL) onto the electrodes which were then allowed to dry for 30 minutes under ambient conditions.

Completed sensors were soaked in 4.4mM potassium phosphate buffer solution (PBS) at pH 7.0 for a minimum of 1 hour to reduce current drift and impregnate the sol-gel with buffer solution. Sensors were then rinsed with UP H_2O, soaked in fresh PBS for an additional 30 minutes, then rinsed again. All biosensors were subjected to +0.65V in 25mL of fresh PBS for 1h to stabilize sensor reading prior to injection of lactate. Sensors were calibrated by adding successive injections of sodium lactate (25µL; 1M) to reach a buffer-lactate concentration of 10mM.

Wire Electrodes. For the lactate biosensors on wires, a polypyrrole layer was first applied to assist the selectivity of the sensors. A solution of pyrrole was prepared (25mL, 0.1M) with 0.1mM H_2SO_4, degassed with N_2 for 20 minutes and electropolymerized onto the platinum wires while stirring using cyclic voltammetry (0.0 to +0.9V, 5 cycles, 50mVs^{-1}). Sensors were allowed to dry for 45 minutes at 50% relative humidity before being set with sol-gels.

Sensors were then coated with two layers of a lactate oxidase (LOx) and bovine serum albumin (BSA) aqueous solution (0.8mg of LOx and 0.8mg of BSA in 75µL of UP H_2O) using a dip-coating procedure (15 dips, 5s submerged time, 15s dry period under ambient conditions) with a 30 minute dry time at 50% RH following each of the two layers. Co-doped sol-gels of HMTES were formed by vortexing 10µL of HMTES with 40µL of NaOH (0.1M) for 10 minutes, then adding 20µL of the aforementioned aqueous enzyme solution. The vial was sealed and machine-vortexed for an additional 10 minutes to facilitate the formation of a sol-gel. Sensors were then coated with two layers of sol-gel using a dip-coating procedure (15 dips, 5s submerged time, 15s dry period under ambient conditions) with a 30 minute dry time at 50% RH between the two layers. Sol-gel coated electrodes were allowed to form xerogels over 24h at 50% RH.

An outer layer of polyurethane (PU) was then deposited in order to limit diffusion and improve selectivity. PU was prepared by adding 100mg of HPU to a 5mL solution of THF/EtOH (50:50 v/v) and stirred overnight. Sensors were dip-coated (10 dips, 5s submerged time, 15s dry period under ambient conditions) then allowed to dry for 30 minutes at 50% RH. The tip of the sensor was capped with epoxy and allowed to dry for 45 minutes at 50% RH, resulting in a 3-4 mm sensing pseudo-cavity.

Completed sensors were soaked in 60mM potassium phosphate buffer solution (PBS) at pH 7.4 for a minimum of 1 hour to reduce current drift and impregnate the sol-gel with buffer solution. Sensors were then rinsed with UP H_2O, soaked in fresh PBS for an additional 30 minutes, then rinsed again. All biosensors were subjected to +0.65V in 25mL of fresh PBS for 1h to stabilize sensor reading prior to injection of lactate. Sensors were calibrated by adding successive injections of sodium lactate (25µL; 0.2M) to reach a buffer-lactate concentration of 6mM.

Xanthine Sensor Fabrication and Performance **(wire and macroelectrodes)**

Macroelectrodes. For the xanthine biosensors on wires, xanthine oxidase (XOx) doped sol-gels of PTMS were formed by first dissolving 0.7 mg of XOx in 75 μL of H_2O in a centrifugation vial and, in a separate vial, diluting 25 μL of PTMS with 100 μL of tetrahydrofuran (THF). These vials were sealed and hand-vortexed vigorously for 5 minutes and 1 minute, respectively. After individual mixing, 50 μL of the XOx/H_2O solution was transferred to the PTMS/THF mixture and shaken for an additional 1 minute to facilitate the formation of a sol−gel. Electrodes were coated by dropcasting 3μL of the resulting sol-gel onto the Pt electrode surface. Sol-gel coated electrodes were allowed to form xerogels over 24h at 50% RH.

An outer polyurethane (PU) layer was then deposited in order to limit diffusion and improve xanthine selectivity. [Freeman 2013; 34,35; Schoenfisch 2016] PU was prepared by adding 100 mg of HPU to a 5 mL solution of THF/EtOH (50:50 v/v) and stirred overnight. The PU was dropcast (10μL) onto the electrodes which were then allowed to dry for 30 minutes under ambient conditions.

Completed sensors were soaked in 10 mM potassium phosphate buffer solution (PBS) at pH 7.0 for a minimum of 1 h to reduce current drift and impregnate the sol−gel with buffer solution. All biosensors were subjected to +0.35 V in 25 mL of PBS for 20 min prior to injection of any xanthine to stabilize sensor reading. Sensors were calibrated by adding successive injections of xanthine (25 μl; 10 μM) to reach a buffer-xanthine concentration of 100 μM.

Wire electrodes. For the xanthine biosensors on wires, xanthine oxidase (XOx) and bovine serum albumin (BSA) co-doped sol-gels PTMS were formed by first dissolving 0.7 mg of XOx and 0.8 mg of BSA in 75 μL of H_2O in centrifugation vials and, in a separate vial, diluting 25 μL of PTMS with 100 μL of tetrahydrofuran (THF). These vials were sealed and hand-vortexed vigorously for 5 minutes and 1 minute, respectively. An enzyme underlayer was deposited onto the sensor via dip-coating in the XOx/BSA/H_2O solution 5 times (5 s submerged time, 15 s dry period under ambient conditions), and allowed to dry for 30 minutesat 50% RH. To formulate the sol-gel, 50 μL of the XOx/BSA/H_2O solution was transferred to the PTMS/THF mixture and shaken for an additional 1 minute. The process of deposition of the sol-gel onto the wire electrode involved coating with the XOx precursor solution by dip-coating the sensor 20 times (5 s submerged time, 15 s dry period under ambient conditions). The electrodes were then dried horizontally for 30 minutes at 50% RH before a second sol–gel deposition. The second layer of enzyme-containing-sol–gel was prepared as above, following the same dip-coating procedure. Sol−gel coated electrodes were allowed to form xerogels over 24 h at 50% RH.

An outer polyurethane (PU) layer was then deposited in order to limit diffusion and improve xanthine selectivity. [Freeman 2013; 34,35; Schoenfisch 2016] PU was prepared by adding 100 mg of HPU to a 5 mL solution of THF/EtOH (50:50 v/v) and stirred overnight. The PU was dip-coated on the wire electrodes for 5 cycles as described above and were allowed to dry for 30 minutes horizontally at 50% RH. The tip of the sensor was capped with epoxy and allowed to dry for 45 minutes at 50% RH, resulting in a 3-4 mm sensing pseudo-cavity.

Completed sensors were soaked in 60 mM potassium phosphate buffer solution (PBS) at pH 7.4 for a minimum of 1 h to reduce current drift and impregnate the sol−gel with buffer solution. All biosensors were subjected to +0.35 V in 25 mL of PBS for 20 min prior to injection of any xanthine to stabilize sensor reading. Sensors were calibrated by adding successive injections of xanthine (25 μl; 10 μM) to reach a buffer-xanthine concentration of 100 μM.

Uric Acid Sensor Fabrication and Performance **(wire and macroelectrodes)**

For the uric acid biosensors on wire electrodes, urate oxidase or uricase (UOx) doped sol-gels of HMTES were formed by first dissolving 4.0 mg of UOx in 75 μL of NaOH (0.1 M) in a centrifugation vial and, in a separate vial, diluting 25 μL of HMTES with 100 μL of tetrahydrofuran (THF). These vials were sealed and hand-vortexed vigorously for 5 minutes and 1 minute, respectively. After individual mixing, 50 μL of the UOx/NaOH solution was transferred to the HMTES/THF mixture and shaken for an additional 1 minute to facilitate the formation of a sol−gel. The process of deposition of the sol-gel onto the wire electrode involved coating with the UOx precursor solution by dip-coating the sensor 5 times (5 s submerged time, 10 s dry period under ambient conditions). The electrodes were then dried horizontally for 30 minutes at 50% RH before the next sol–gel deposition. The second layer of sol–gel was prepared as above with the omission of UOx, following the same dip-coating procedure to provide a diffusion-limiting layer. Sol−gel coated electrodes were allowed to form xerogels over 48 h at 50% RH.

After application of the sol-gel layers, a 1:10 polyluminol:polyaniline (PL-A) layer was applied to assist the selectivity of the sensors.[Chen 2016; Conway 2016;38] A solution of luminol was prepared (25 mL, 0.5 mM) with 5 mM H_2SO_4 (0.1 M), degassed with N_2 for 20 minutes and electropolymerized electropolymerized onto the wire sensor while stirring using cyclic voltammetry (0.0 to +1.0 V, 12 cycles, 50 mVs^{-1}). Sensors were rinsed with UP H_2O and allowed to dry horizontally for 30 minutes at 50% RH. An outer polyurethane (PU) layer was then deposited in order to limit diffusion and improve uric acid selectivity. [Freeman 2013; 34,35; Schoenfisch 2016] PU was prepared by adding 100 mg of HPU to a 5 mL solution of THF/EtOH (50:50 v/v) and stirred overnight. The PU was dip-coated on the wire electrodes for 10 cycles as described above and were allowed to dry for 30 minutes horizontally at 50% RH. The tip of the sensor was capped with epoxy and allowed to dry for 45 minutes at 50% RH, resulting in a 3-4 mm sensing pseudo-cavity.

Completed sensors were soaked in 60 mM potassium phosphate buffer solution (PBS) at pH 7.4 for a minimum of 1 h to reduce current drift and impregnate the sol−gel with buffer solution. All biosensors were subjected to +0.65 V in 25 mL of PBS for 20 min prior to injection of any uric acid to stabilize sensor reading. Sensors were calibrated by adding successive injections of uric acid (50 μl; 50 mM) to reach a buffer-uric acid concentration of 400 mM.

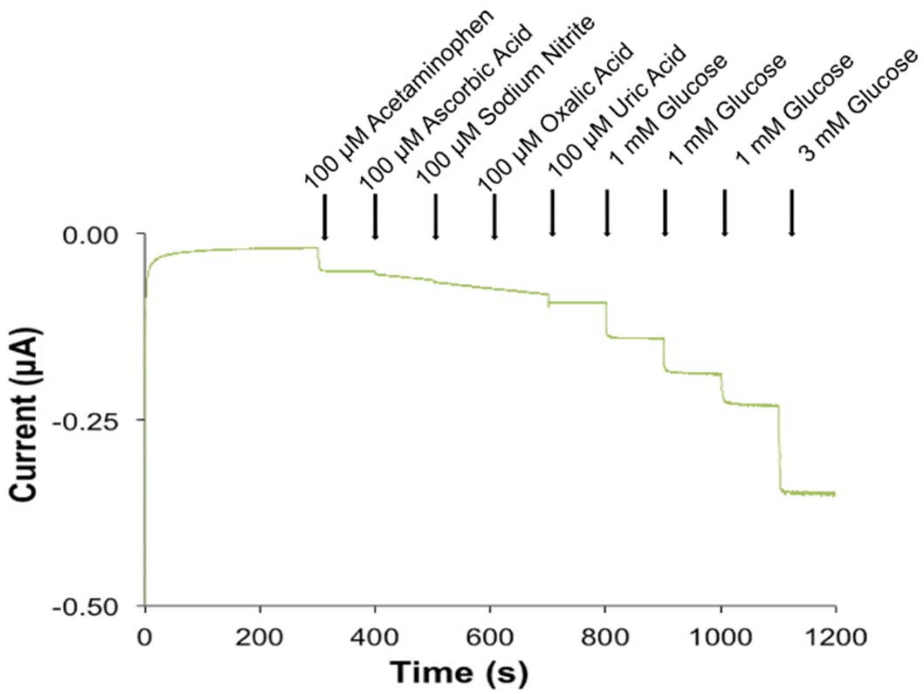

Figure SM-1. Amperometric I-t curves during successive injections of common interferent species (100 µM) and glucose (1 mM and/or 3mM) at a platinum wire (diameter of 127 µm; 203 µm PTFE-coated) electrode modified with GOx-doped OTMS xerogel, undoped OTMS xerogel, polyphenol (PP), and 50:50 polyurethane layer (PU) without epoxy cap; Solution: 60.0 mM PBS, pH = 7.4, µ = 155 mM.

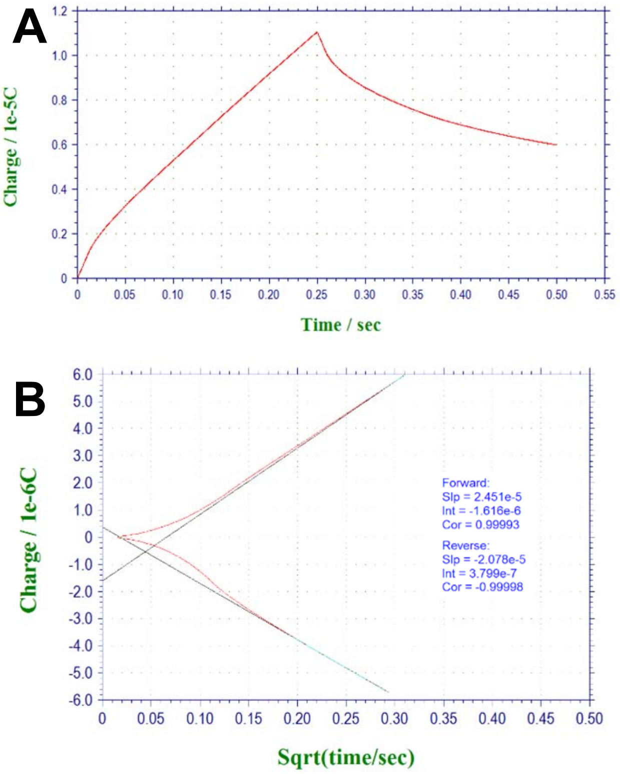

$$Q = 2nFACD^{1/2}\pi^{-1/2}t^{1/2}$$

$$A = \frac{Slope \times \pi^{1/2}}{2nFCD^{1/2}}$$

Figure SM-2. **(A)** Charge vs. time plot for chronocoulometry experiments of 5 mM $K_3Fe(CN)_6$ (0.5 M KCl) where the potential is stepped from a potential with negligible Faradaic current (0 V) to +0.60 V (vs. Ag/AgCl); **(B)** Corresponding Anson plot (charge vs. time^{½}) to determine the area of the electrode (inset equations above).

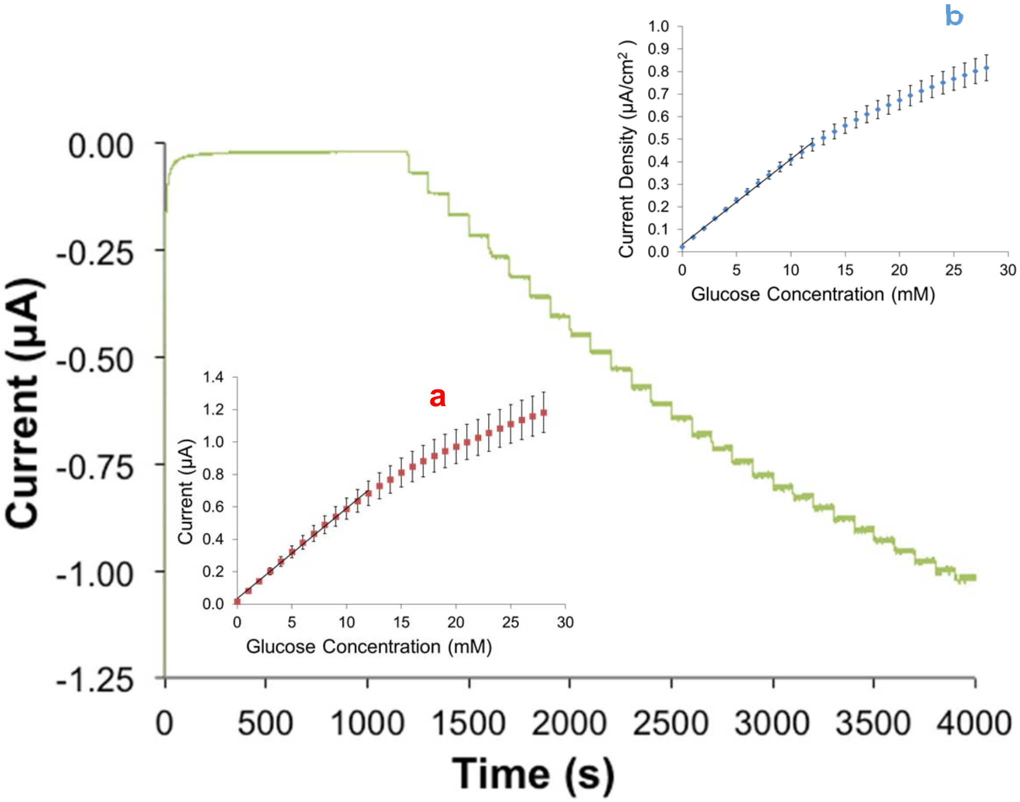

Figure SM-3. Typical amperometric I─t curve, (a) corresponding calibration curves, and (b) corresponding calibration curve standardized to electrode area during successive 1 mM injections of glucose at a platinum-iridium wire electrode modified with GOx-doped OTMS xerogel, undoped OTMS xerogel, polyphenol (PP), 50:50 polyurethane layer (PU), and epoxy cap; Solution: 60.0 mM PBS, pH = 7.4, μ = 155 mM. The linear range is from 1-12 mM of glucose. Note: The error bars represent standard error (n≥8).

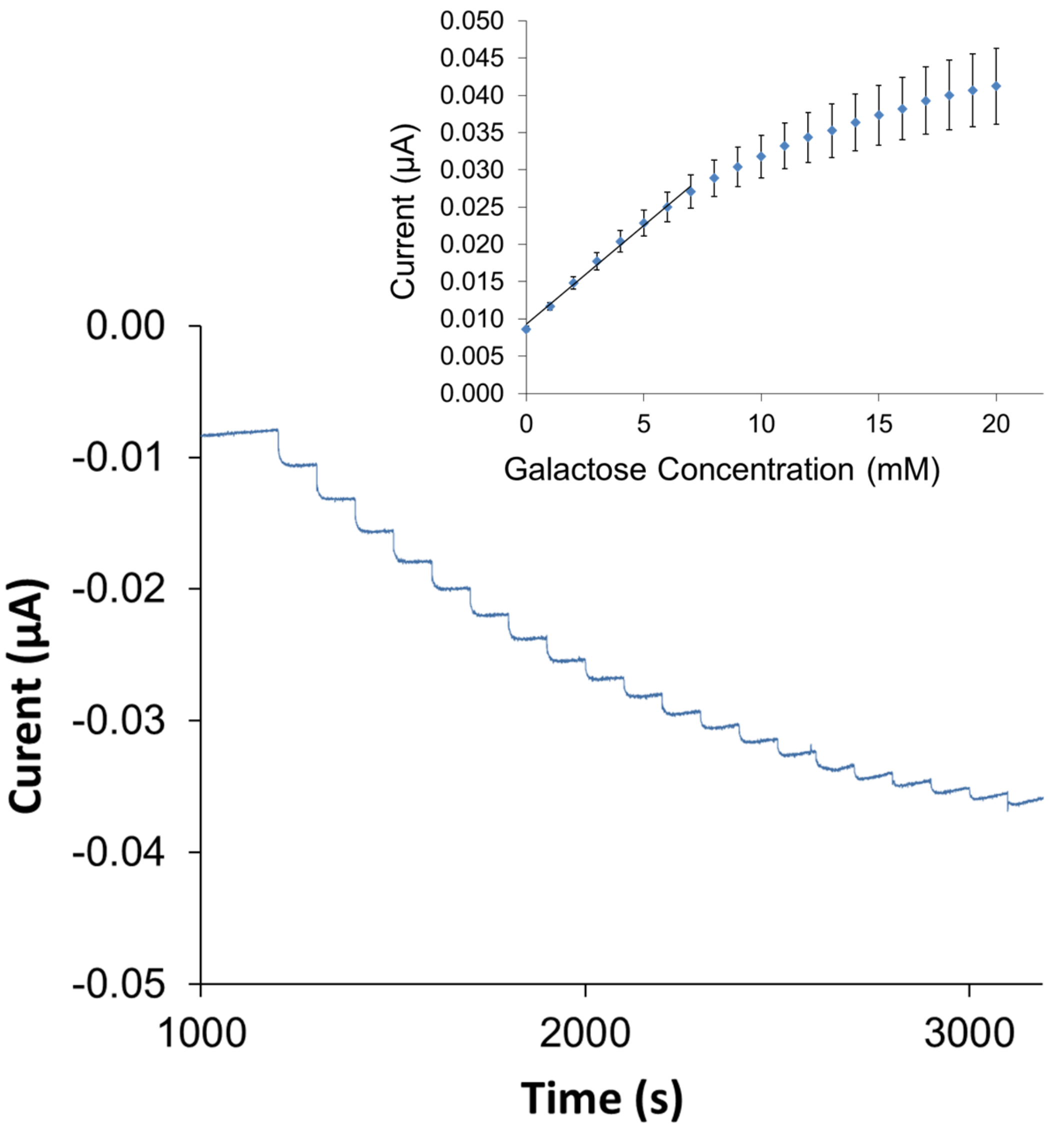

Figure SM-4. Typical amperometric I–t response and calibration curve (inset) during successive 1 mM injections of galactose at a Pt-Ir wire electrode modified with GaOx-doped IBTMS xerogel, 50:50 PU layer, and epoxy cap. Solution: 60.0 mM PBS, pH = 7.4, μ = 155 mM. Note: The error bars represent standard error (n≥6). Solution: 60.0 mM PBS, pH = 7.4, μ = 155 mM.

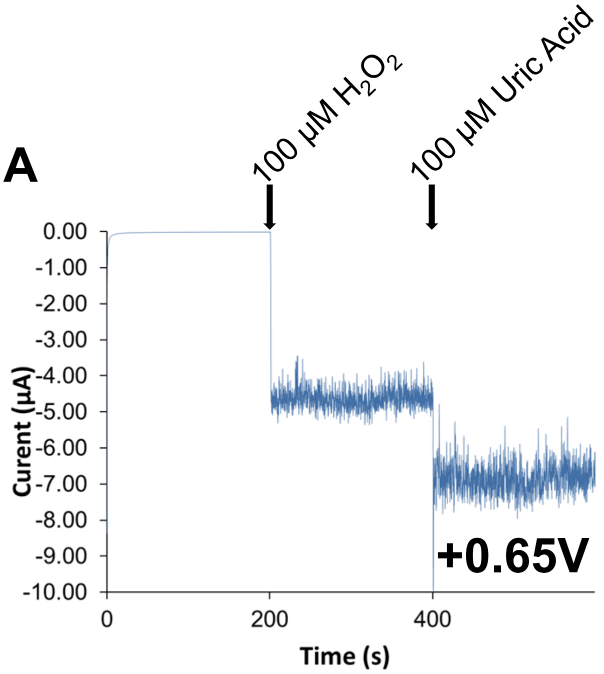

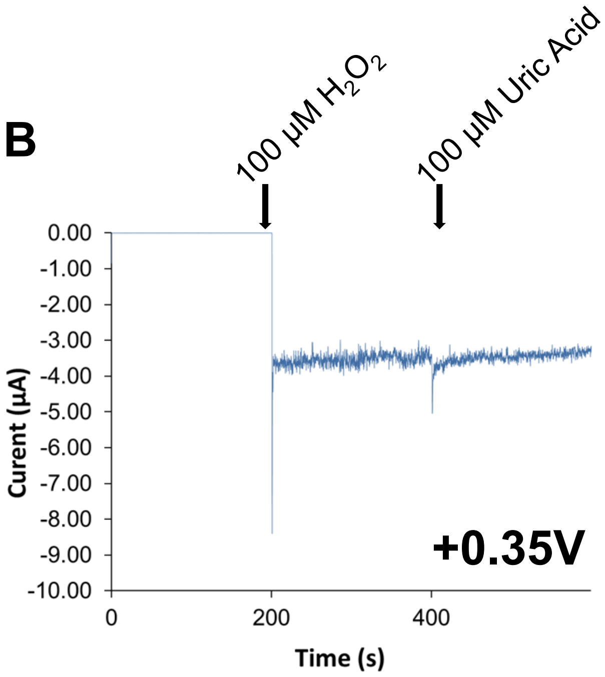

Figure SM-5. **(A)** Amperometric I-t curve showing 100 μM injection of hydrogen peroxide followed by 100 μM injection of uric acid at a bare platinum macroelectrode, held at potentials of +0.65V and +0.35V. Solution= 10.0 mM PBS, pH = 7.0. At +0.65 V the signal decreases with addition of both hydrogen peroxide and uric acid. At +0.35V the signal does not respond significantly with the injection of uric acid, suggesting that the presence of uric acid is not detected at this potential.

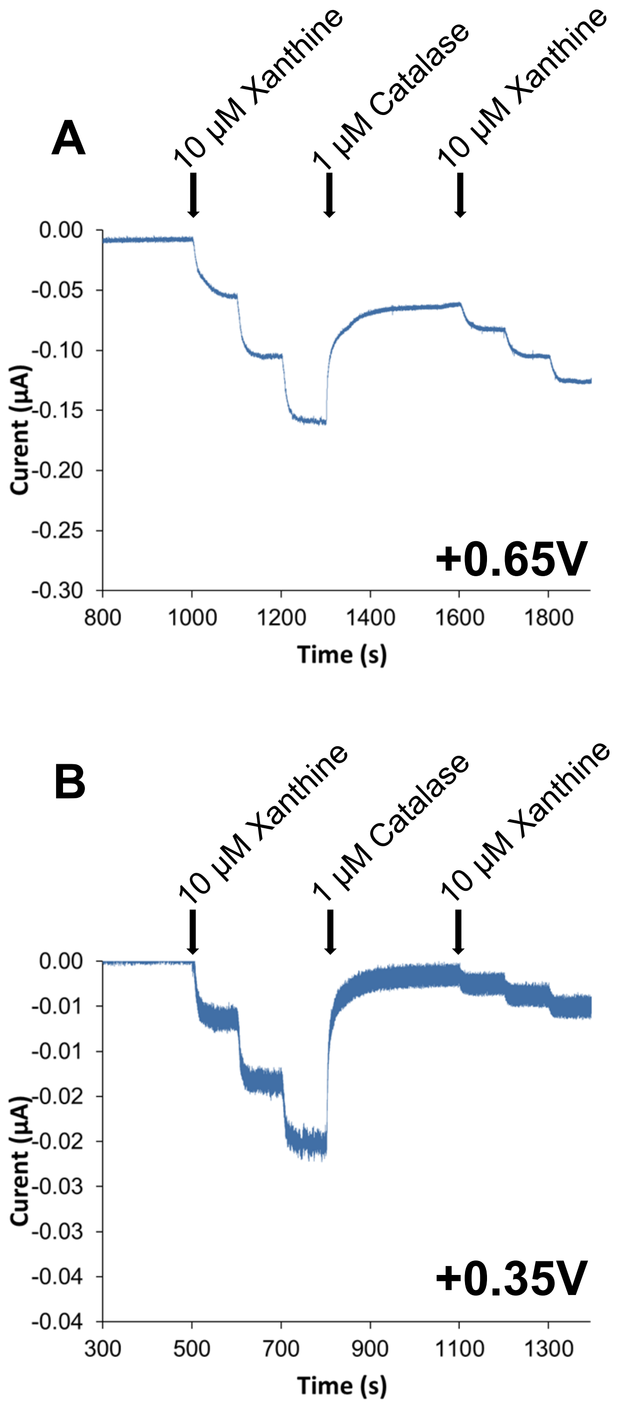

Figure SM-6. Amperometric I-t curve during successive 10 µM injections of xanthine followed by 1µM injections of catalase and 10 µM xanthine at a platinum macroelectrode modified with XOx-doped PTMS xerogel and polyurethane layer (100% HPU), held at potentials of **(A)** +0.65V and **(B)** +0.35V. Note: Solution conditions - 10.0 mM PBS, pH = 7.0.

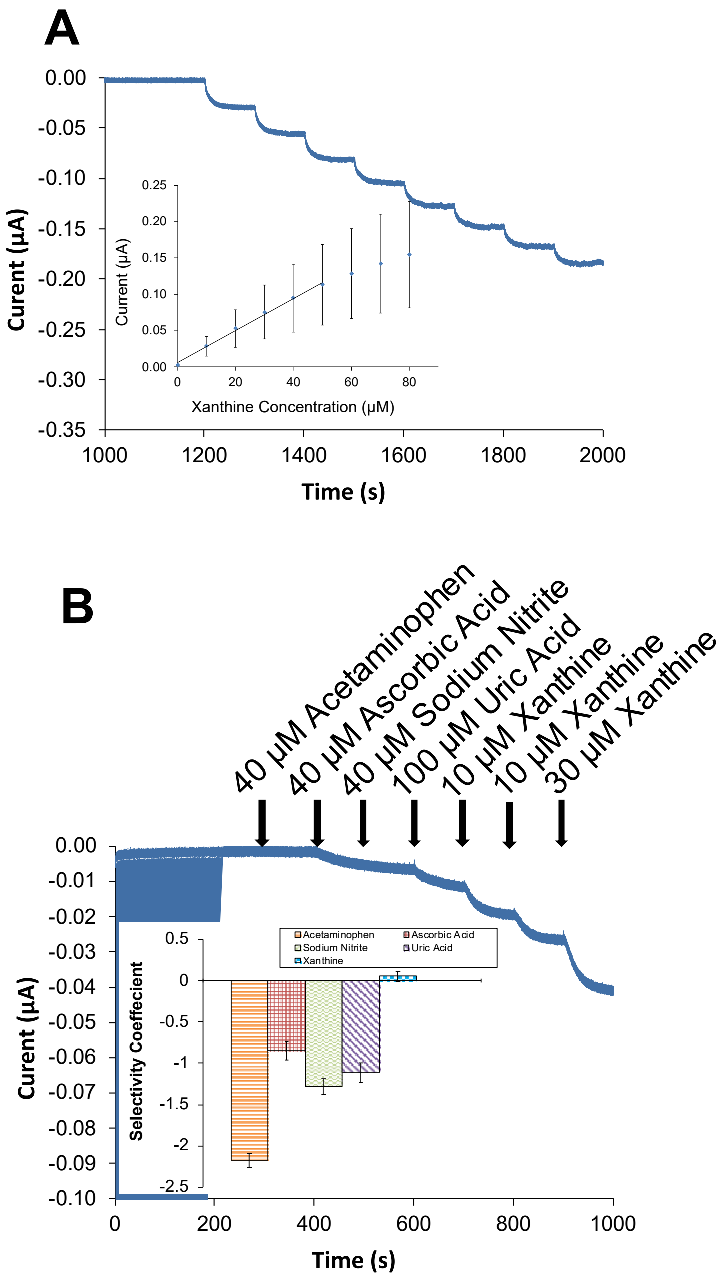

Figure SM-7. (A) Typical amperometric I–t response and calibration curve (inset) during successive 10 µM injections of xanthine and; **(B)** Amperometric I-t response and calculated selectivity coefficients (inset) during successive injections of common interferent species (40 µM) and xanthine (10 µM and/or 30 µM) at a platinum macroelectrode modified with XOx-doped PTMS xerogel, and polyurethane layer (100% HPU). Notes: 10.0 mM PBS; pH 7; Error bars represent standard error (n≥3).

Table SI-1. Comparison of Xanthine Biosensor Performance Parameters–Literature Comparison.

System	Type	WE	Average Sensitivity (nA/µM)	Response Time† (s)	Linear Range (µM)	LOD‡ (µM)	Stability	Ref
Pt / PTMS (XOx) / 100% HPU	1st G	Pt	2.20	22.5 (± 6.5)	50	1.7 (± 0.2)	-	Current Work
Graphite electrode/XOx suspended in gelatine	1st G	GCE	210	-	40	4.5	35% decrease after 30 h	59
Pt / PPy / XOx	1st G	Pt	-	-	1000	-	39% decrease after 120 d	60
CPE / MWCNT-XOx	1st G	CPE	-	150	100	-	-	61
Pt / PANI / MWCNT / XOx	1st G	Pt	-	4	58	0.6	50% loss over 200 uses, 100 d	62
GCE / MWCNT / XOx	1st G	Pt	-	5	6	0.08	6% loss in 12 d	63
Au / Poly-TTCA / XOx	1st G	Au	5.35	10	100	1.0	-	64
GCE / CHT / XOx / GTD / KH	1st G	GCE	6.96	-	18	0.0215	20 d	65

Notes: Uncertainty values represent standard error (n=5); †Determined as time to 95% of total amperometric response; ‡LOD = $3\sigma_{blank}/b_1$; Pt: platinum; XOx: Xanthine oxidase; PTMS: n-propyltrimethoxysilane; HPU: hydrothane polyurethane; Ppy: polypyrrole; CPE: carbon paste electrode; MWCNT: multi-walled carbon nanotubes; PANI: polyaniline; GCE: glassy carbon electrode; Poly-TTCA: Poly-5, 2′: 5′, 2″-terthiophine-3-carboxylic acid; CHT: chitosan; GTD: glutaraldehyde; KH: 3-aminopropyltriethoxysilane.

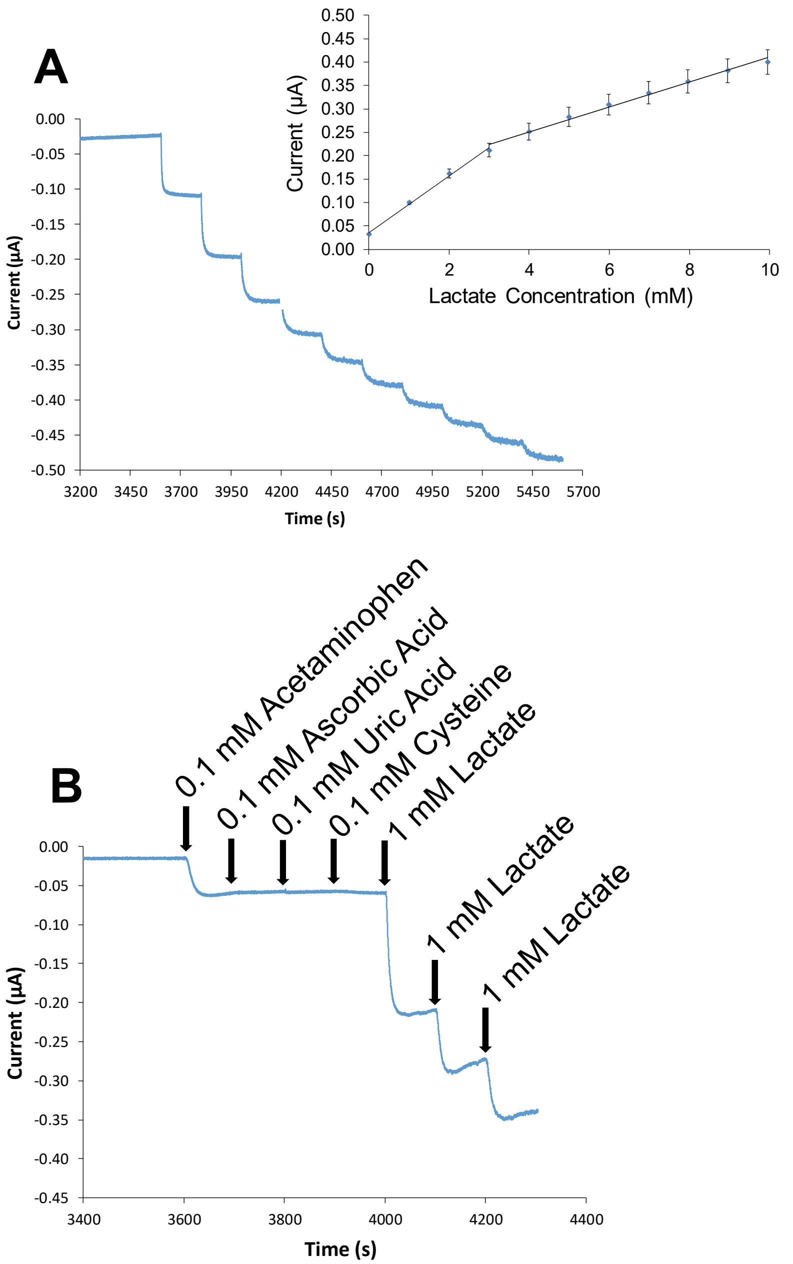

Figure SM-8. (A) Typical amperometric I−t response and calibration curve (inset) during successive 1 mM µM injections of sodium lactate and; **(B)** Amperometric I-t response and calculated selectivity coefficients (inset) during successive injections of common interferent species (0.1 mM) and sodium lactate (1 mM) at a platinum macroelectrode modified with polymerized pyrrole, LOx-doped HMTES xerogel, and polyurethane layer (100% HPU). Notes: 10.0 mM PBS; pH 7; Error bars represent standard error (n≥3).

Table SI-2. Comparison of Lactate Biosensor Performance Parameters–Literature Comparison.

System	Type	WE	Sensitivity (nA/µM)	Response Time† (s)	Linear Range (mM)	LOD‡ (µM)	Stability	Ref
Pt / PPY / HMTES (w/LOx +BSA) / 100% HPU	1st G	Pt	0.0268	27.4 (±7.5)	3 - 10	29.5	-	Current Work
Pt / PPYox / glutaraldehyde co-crosslinking of LOx w/ BSA	1st G	Pt	0.0072	-	0 - 20	-	-	72
ITO glass / PANI-co-FlANI / glutaraldehyde coupling w/ LOx	1st G	ITO/glass	1.18	-	0.1 - 5.5	100	no loss of activity after 150 uses over 26 days	73
Au / LOx-DSTP	1st G	Au	0.69	-	0 - 0.2	40	50% decrease after one use then constant for 1 month	74
GCE / TEOS + LOx / MWCNTs	1st G	GCE	6.031	-	0.2 - 2.0	0.3	10% decrease after 4 weeks of testing (once/5 days)	75
Pt / 3-APTMS + EETMS / LOx	1st G	Pt	-	-	0.05 - 5	10	stable over 30 days (700 injections)	39
Pt / albumin + mucin + glutaraldehyde coupling w/ LOx	1st G	Pt	0.79	90 (for 100% response)	0.0007 - 1.5	0.7	13% decrease over 27 days (2 tests/week)	76
Au glass / Nafion / TNTs + LOx	1st G	Au/glass	0.24	5 (for 90% response)	0.5 - 14	200	-	77
Au / MPTS + LOx + AuNPs	1st G	Au	3.4	-	0.05 - 0.25	4	-	78
GCE / Nafion / LOx / N-CNTs	1st G	GCE	40	-	0.014 - 0.325	4.1	-	79
GCE / LOx + sol-gel / Pt NPs / CNTs	1st G	GCE	6.4	-	0.2 - 2	0.3	-	80

Notes: Uncertainty values represent standard error (n=5); †Determined as time to 95% of total amperometric response; ‡LOD = $3\sigma_{blank}/b_1$; Pt: platinum; PPY: polypyrrole; LOx: Lactate oxidase; HMTES: hydroxymethyltriethoxysilane; BSA: bovine serum albumin; HPU: hydrothane polyurethane; PPYox: overoxidized polypyrrole; ITO: indium tin oxide; PANI-coFlANI: poly(aniline-co-fluoroaniline); Au: gold; DTSP: 3,3' dithiodipropionic acid di(*N*-succinimidyl ester); GCE: glassy carbon electrode; TEOS: Tetra-ethyl Orthosilicate; MWCNT: multi-walled carbon nanotubes; APTMS: (3-aminopropyl)trimethoxysilane; EETMS: 2-(3,4-epoxycyclohexyl)ehtyltrimethoxysilane; TNT: titanate nanotubes; MPTS: (3-mercaptopropyl)-trimethoxysilane; N-CNT: nitrogen-doped carbon nanotubes; NP: nanoparticles.

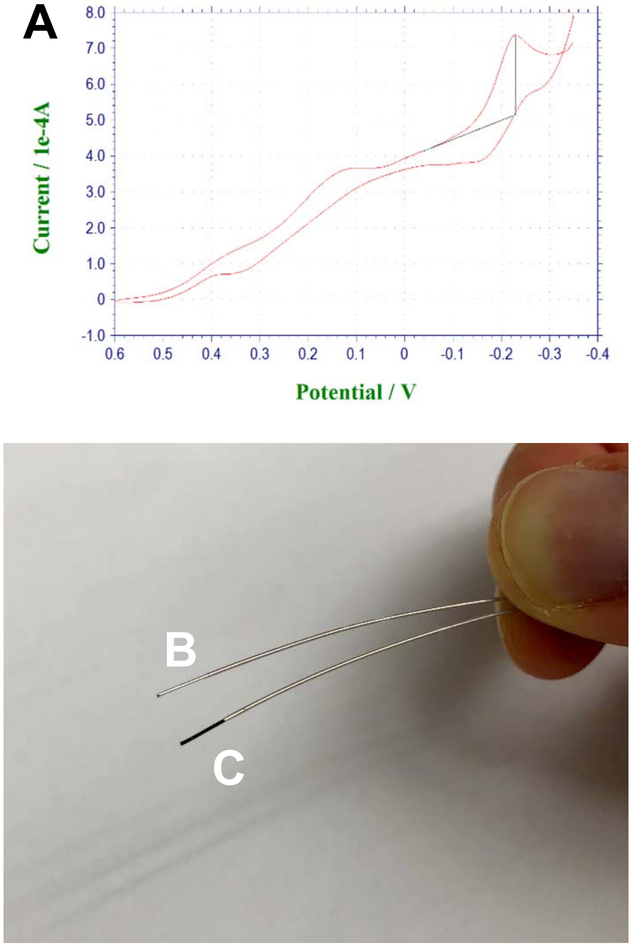

Figure SM-9. (A) Cyclic voltammetry during platinization to form platinum black at a clean platinum electrode; solution is 3% chloroplatinic acid (v/v in water) by cycling the potential from +0.6 to −0.35 V (vs Ag/AgCl) at sweep rate of 20 mV/s; Photographs of platinum-iridium wire electrode **(A)** before and **(B)** after application of platinum black modification.

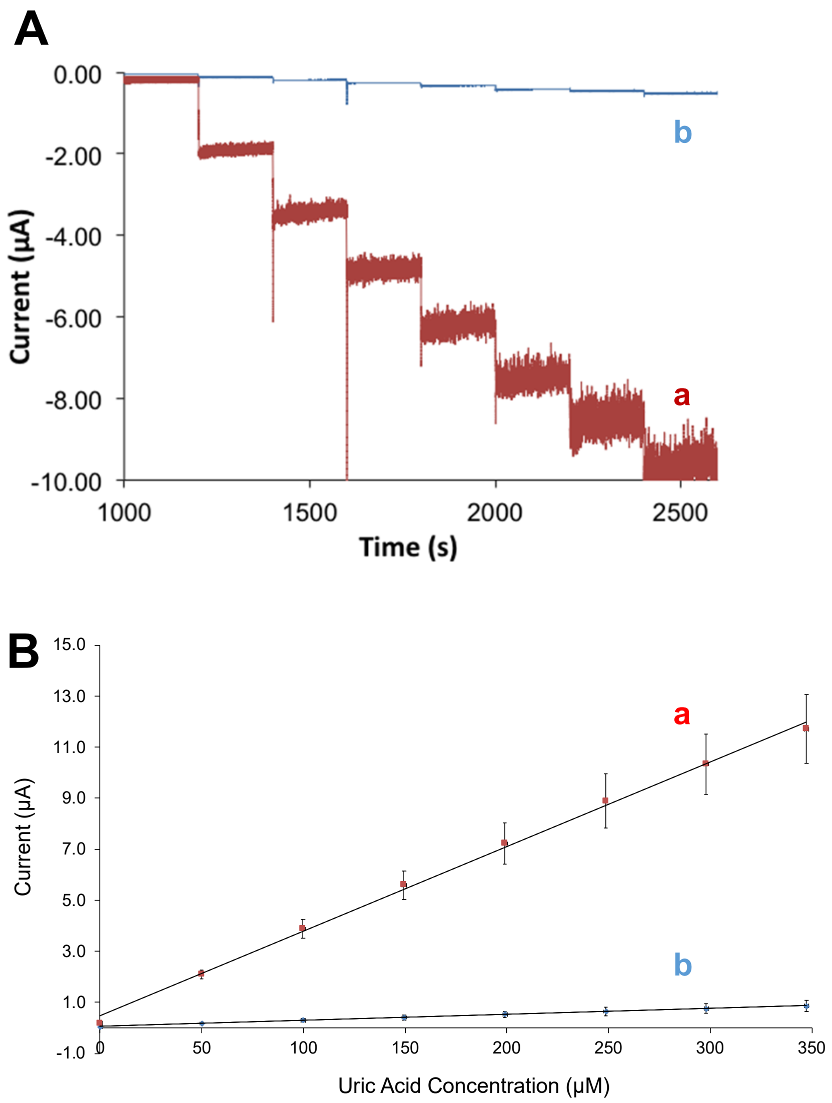

Figure SM-10. **(A)** Amperometric I-t response and **(B)** corresponding calibration curves during successive 100 µM injections of uric acid at a Pt-Ir wire electrode modified with UOx-doped HMTES xerogel, undoped HMTES xerogel, polyluminol-polyaniline (PL-A), and polyurethane layer (100% HPU), and epoxy cap both **(a)** with and **(b)** without a Pt-black underlayer. Solution: 60.0 mM PBS, pH = 7.4, µ = 155 mM. Note: The error bars represent standard error (n≥6).

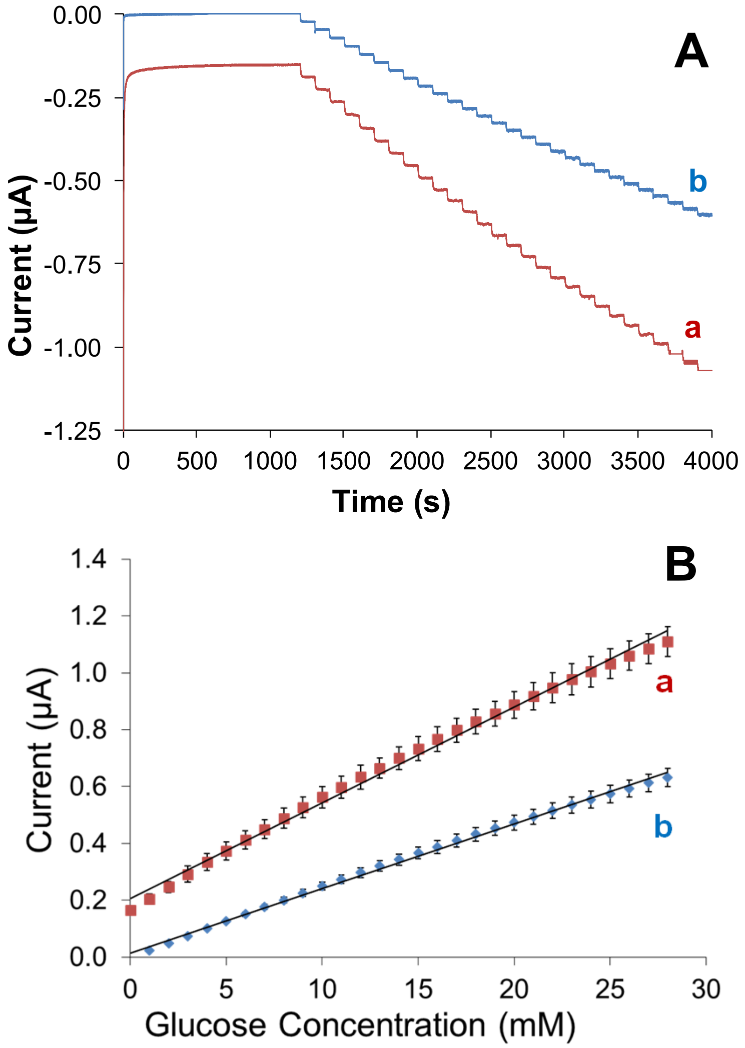

Figure SM-11. (A) Amperometric I-t curve and (B) corresponding calibration curves during successive 1 mM injections of glucose at a platinum macroelectrode modified with GOx-doped OTMS xerogel, undoped OTMS xerogel, polyphenol (PP), and polyurethane layer (PU) in **(a)** blood serum and **(b)** synthetic urine. Note: The error bars represent standard error ($n \geq 3$).

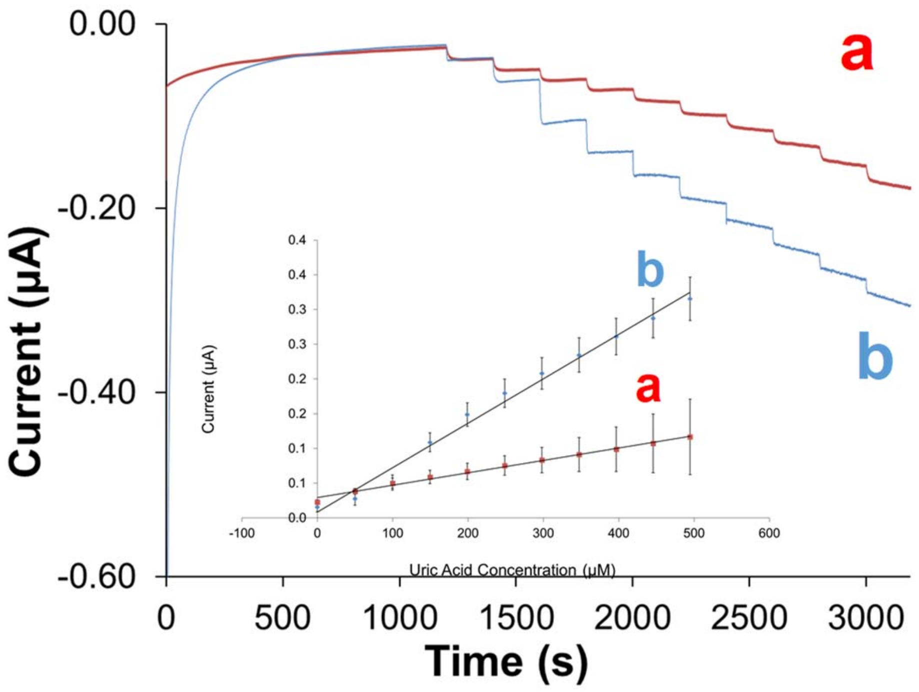

Figure SM-12. Amperometric I-t response and corresponding calibration curves (inset) during successive 100 µM injections of uric acid at a platinum macroelectrode modified with UOx-doped HMTES xerogel, undoped HMTES xerogel, polyluminol-polyaniline (PL-A), and polyurethane layer (100% HPU) in **(a)** blood serum and **(b)** synthetic urine. Note: The error bars represent standard error (n ≥ 6).

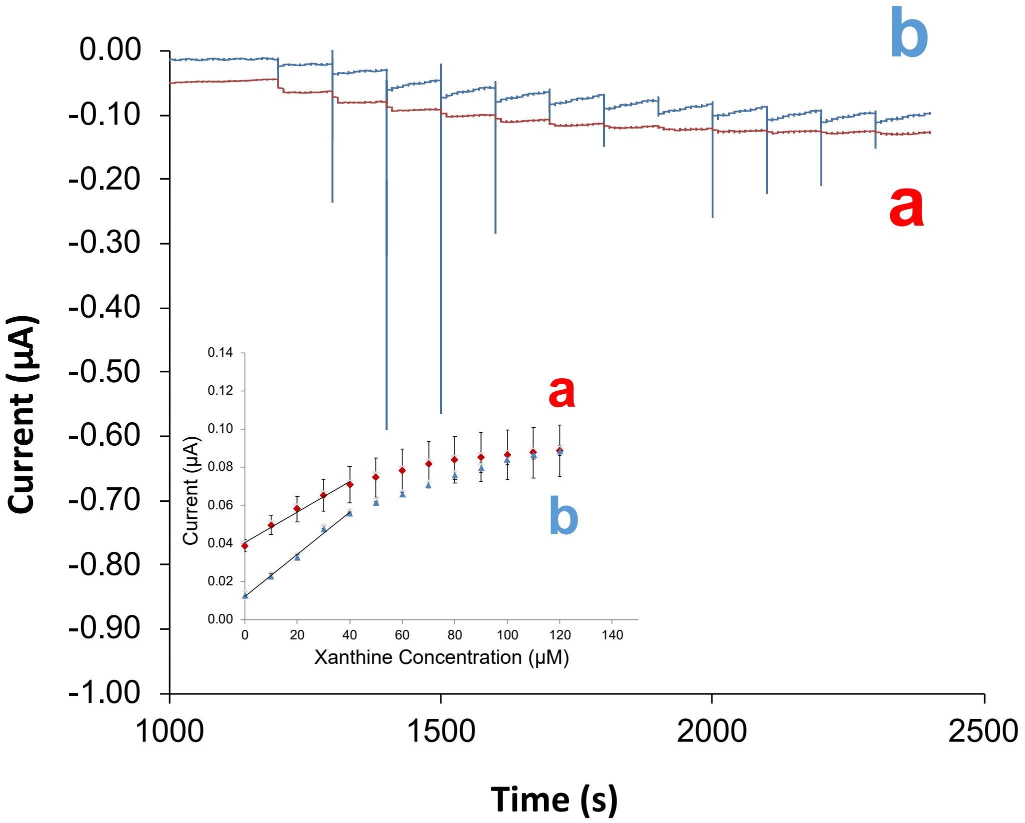

Figure SM-13. Amperometric I-t response and corresponding calibration curves (inset) during successive 100 µM injections of xanthine at a platinum macroelectrode modified with XOx-doped PTMS xerogel and polyurethane layer (100% HPU) in **(a)** blood serum and **(b)** synthetic urine. Note: The error bars represent standard error ($n \geq 5$).

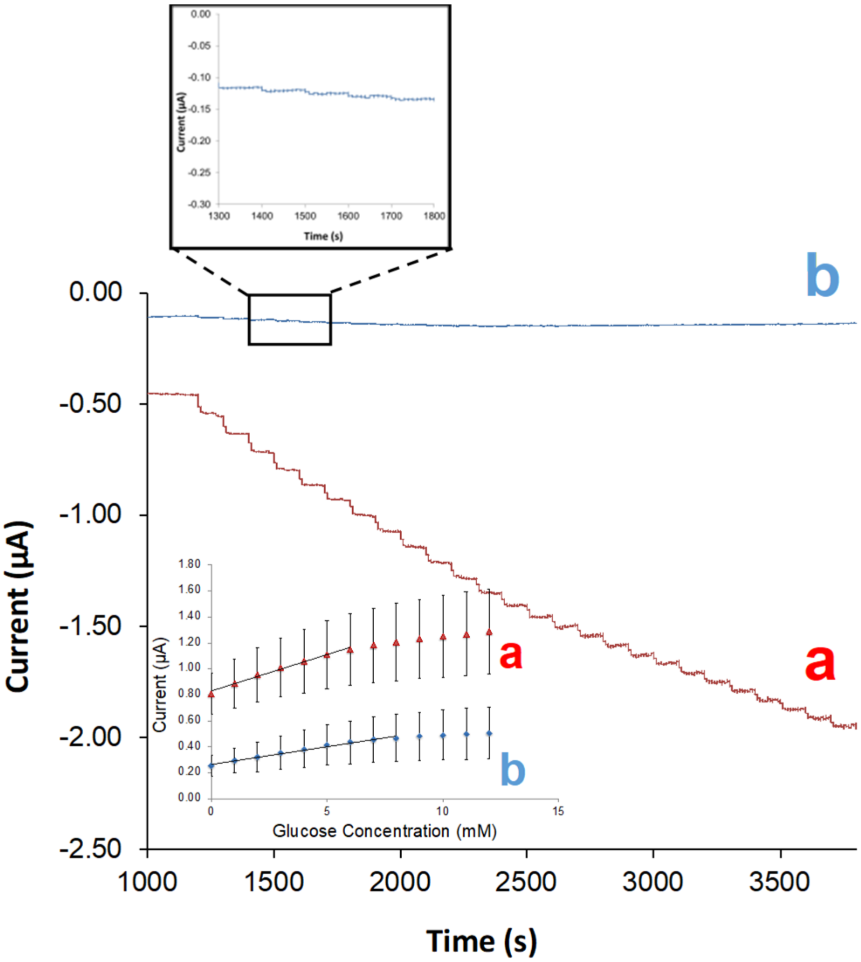

Figure SM-14 Amperometric I−t curve and calibration curve during successive 1 mM injections of glucose at a platinum-iridium wire electrode modified with (a) GOx-doped OTMS xerogel, undoped OTMS xerogel, polyphenol (PP), and polyurethane layer (50% HPU/TPY), and epoxy cap both **(a)** with and **(b)** without a Pt-black underlayer; Solution: serum.

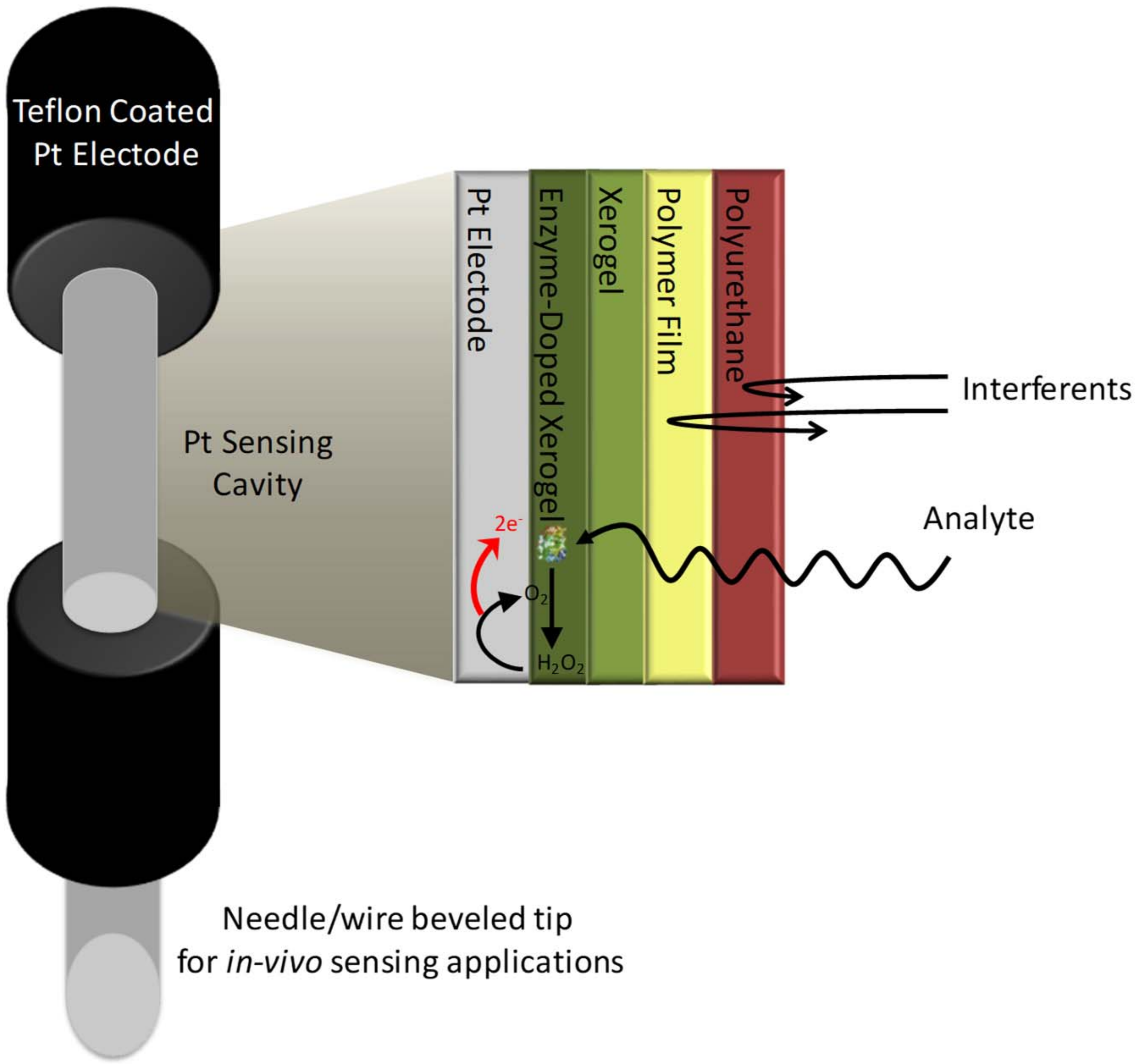

Figure SM-15. Generic illustration of LbL assembly of layered materials including enzyme-doped and undoped xerogel, electrochemically deposited pyrrole polymer (for lactate sensing), and polyurethane layers in a sensing cavity of a beveled needle for *in-vivo* sensing applications (e.g., continuous monitoring of lactate sepsis diagnosis/monitoring in an emergency room situation). Sensors could be inserted along with an intravenous line.
